# Development and Evaluation of Competitive Inhibitors of Trastuzumab-HER2 Binding to Bypass the Binding-Site Barrier

**DOI:** 10.3389/fphar.2022.837744

**Published:** 2022-02-18

**Authors:** Brandon M. Bordeau, Lubna Abuqayyas, Toan D. Nguyen, Ping Chen, Joseph P. Balthasar

**Affiliations:** Department of Pharmaceutical Sciences, School of Pharmacy and Pharmaceutical Sciences, University at Buffalo, Buffalo, NY, United States

**Keywords:** binding site barrier, ADC tumor pharmacokinetics/pharmacodynamics, T-DM1, modeling and simulation, antibody

## Abstract

Our group has developed and experimentally validated a strategy to increase antibody penetration in solid tumors through transient inhibition of antibody-antigen binding. In prior work, we demonstrated that 1HE, an anti-trastuzumab single domain antibody that transiently inhibits trastuzumab binding to HER2, increased the penetration of trastuzumab and increased the efficacy of ado-trastuzumab emtansine (T-DM1) in HER2+ xenograft bearing mice. In the present work, 1HE variants were developed using random mutagenesis and phage display to enable optimization of tumor penetration and efficacy of trastuzumab-based therapeutics. To guide the rational selection of a particular 1HE mutant for a specific trastuzumab-therapy, we developed a mechanistic pharmacokinetic (PK) model to predict within-tumor exposure of trastuzumab/T-DM1. A pharmacodynamic (PD) component was added to the model to predict the relationship between intratumor exposure to T-DM1 and the corresponding therapeutic effect in HER2+ xenografts. To demonstrate the utility of the competitive inhibition approach for immunotoxins, PK parameters specific for a recombinant immunotoxin were incorporated into the model structure. Dissociation half-lives for variants ranged from 1.1 h (for variant LG11) to 107.9 h (for variant HE10). Simulations predicted that 1HE co-administration can increase the tumor penetration of T-DM1, with inhibitors with longer trastuzumab binding half-lives relative to 1HE (15.5 h) further increasing T-DM1 penetration at the expense of total tumor uptake of T-DM1. The PK/PD model accurately predicted the response of NCI-N87 xenografts to treatment with T-DM1 or T-DM1 co-administered with 1HE. Model predictions indicate that the 1HE mutant HF9, with a trastuzumab binding half-life of 51.1 h, would be the optimal inhibitor for increasing T-DM1 efficacy with a modest extension in the median survival time relative to T-DM1 with 1HE. Model simulations predict that LG11 co-administration will dramatically increase immunotoxin penetration within all tumor regions. We expect that the mechanistic model structure and the wide range of inhibitors developed in this work will enable optimization of trastuzumab-cytotoxin penetration and efficacy in solid tumors.

## 1 Introduction

There is substantial interest in the development of strategies to increase monoclonal antibody (mAb) uptake and distribution in solid tumors (Bordeau and Balthasar, 2021). The uptake and penetration of mAb within tumors is limited due to several pathophysiological characteristics and biological phenomena associated with tumors, including high tumor interstitial fluid pressure, dense extracellular matrix development, an abnormal vasculature network, and by the “binding site barrier” (BSB) (Jain and Baxter, 1988; Netti et al., 2000; Pluen et al., 2001; Bordeau and Balthasar, 2021). The BSB was first described in 1990 by Fujimori et al. (1990), Juweid et al. (1992) and has since been characterized through experimental investigations and through application of mathematical modeling and simulation (Saga et al., 1995; Thurber et al., 2007; Thurber et al., 2008; Thurber and Wittrup, 2008; Lee and Tannock, 2010; Thurber and Weissleder, 2011a; Thurber and Weissleder, 2011b). Briefly, following extravasation, mAb rapidly binds to cellular antigens, concentrating antibodies at sites near blood vessels and decreasing the extent of within-tumor mAb distribution. Due to this barrier, high-affinity antibodies demonstrate heterogenous distribution within tumors, with high concentrations of antibody at sites near tumor capillaries, and with little or no antibody distribution to distant sites (i.e., >20 µm from tumor capillaries). Recently, there has been renewed interest in the BSB due to the apparent impact of poor tumor penetration on the anti-tumor effects of mAb-cytotoxin conjugates. Radioimmunoconjugates with alpha-emitting radionuclides can kill a cancer cell with 1–10 emissions (Thurber et al., 2007), recombinant immunotoxins (RITs) can achieve cell killing with ∼1,000 bound toxins (Kreitman and Pastan, 1998), and high-potency antibody-drug conjugates (ADCs) may be effective following internalization of <1,000 ADC molecules. Commonly, tumor antigen density exceeds 10^6^ antigens/cell. Heterogenous intra-tumoral ADC distribution, consistent with the BSB, can lead to an overkilling effect for cells in close proximity to blood vessels at the expense of subtherapeutic ADC exposure for the majority of tumor cells which are distant from blood vessels. The BSB can substantially limit therapeutic efficacy, particularly for highly-toxic antibody therapies where the maximum tolerated dose is far below levels needed to saturate the tumor antigen.

Trastuzumab, an anti-HER2 mAb, is currently employed as the targeting vector within two FDA-approved ADCs, ado-trastuzumab emtansine (T-DM1) and fam-trastuzumab deruxtecan-nxki. A third trastuzumab-based ADC, trastuzumab duocarmazine, is in late-stage clinical trials (Rinnerthaler et al., 2019). As the first FDA-approved mAb for solid tumor indications, trastuzumab has been widely used for characterizing the impact of the BSB on mAb and ADC efficacy. For example, using a physiologically-based pharmacokinetic model, Cilliers et al. predicted that, in high HER2 expressing tumors, current clinical doses of T-DM1 result in poor tumor penetration and predicted that the co-administration of unconjugated (i.e., “naked”) mAb with T-DM1 would increase the fraction of tumor cells that are exposed to lethal T-DM1 concentrations (Cilliers et al., 2016; Khera et al., 2018). In an NCI-N87 xenograft mouse model that is insensitive to trastuzumab, co-administration of naked trastuzumab with T-DM1 significantly enhanced T-DM1 efficacy (Cilliers et al., 2018). Similar observations were presented by Singh et al., who employed pharmacokinetic-pharmacodynamic (PK-PD) modeling to demonstrate a synergistic interaction between naked trastuzumab and T-DM1 in NCI-N87 xenograft bearing mice (Singh et al., 2020). Both groups highlighted several limitations of the mAb co-administration strategy including 1) the optimal dose ratio of naked mAb to ADC varies based on tumor antigen expression, 2) ADC binding at smaller metastatic sites may be outcompeted by naked mAb, and 3) tumors with high antigen expression and poor vascularity require grams of naked antibody to effectively saturate tumor antigen, which may not be feasibly administered clinically (Cilliers et al., 2018; Singh et al., 2020).

Our laboratory has developed an alternative method to increase the tumor penetration of anti-tumor antibodies through transient, competition inhibition of mAb-tumor binding. For example, when trastuzumab is engaged with a competitive inhibitor of HER2 binding, the within-tumor distribution of the antibody is not hindered by the BSB. Following dissociation from the inhibitor within the tumor milieu, trastuzumab is free to bind to tumor cell-associated HER2, including at sites distant from blood vessels. In our prior work, an anti-idiotype single domain antibody, 1HE, was employed as a model inhibitor of trastuzumab-HER2 binding (Bordeau et al., 2021). Administration of 1HE with a 2 mg/kg dose of trastuzumab to mice bearing SKOV3 xenografts increased the penetration distance of trastuzumab from tumor vasculature by 40%, and in NCI-N87 xenograft bearing mice, the co-administration of 1HE with a 1.8 mg/kg dose of T-DM1 increased the median time of survival from 29 to 42 days (Bordeau et al., 2021).

Development of competitive inhibitors with a range of binding characteristics may yield optimal agents for application to the varying trastuzumab-based conjugates (ado-trastuzumab emtansine, fam-trastuzumab deruxtecan-nxki, trastuzumab duocarmazine, etc.). In the present work, we take steps to extend the competitive inhibition strategy through the development of 1HE mutants with altered trastuzumab binding half-lives. To support the identification of ideal inhibitors, we developed a semi-mechanistic mathematical model to predict the impact of competitive inhibition on antibody and antibody-conjugate distribution and efficacy.

## 2 Materials and Methods

### 2.1 Antibodies, Mice, and Tumor Cell-Line

Trastuzumab and T-DM1 were purchased from Millard Fillmore Memorial Hospital (Amherst, NY). The gastric carcinoma cell-line NCI-N87 was a generous gift from Dr. Dhaval K. Shah and was authenticated by short tandem repeat profiling by the American Type Culture Collection (ATCC, Manassas, VA) and tested negative for mycoplasma in January of 2021.

### 2.2 Phage Library Construction

One nanogram of 1HE DNA with NdeI and XhoI restriction digestion overhangs was used as the template DNA for error-prone polymerase chain reaction (PCR). PCR primers were designed with sFiI restriction digest overhangs for ligation of the PCR product into the pComb3XSS phagemid plasmid (Addgene, Cambridge, MA). Error-prone PCR product was obtained following 14-cycles of denaturation at 94°C for 1 min, annealing at 52°C for 1 min, and extension at 68°C for 3 min with a final 10-minute extension. PCR product (1 μg) was ligated into the pComb3XSS phagemid (2.4 μg) and transfected into TG1 *E. coli* cells (Lucigen, Middleton, WI) through electroporation. Following electroporation, transformed bacteria were serially diluted up to 10^4^ and spread over lysogeny broth (LB) agar plates supplemented with 2% glucose and 100 μg/ml ampicillin. Remaining transformed bacteria were spread onto four 245 mm square dishes containing LB agar with 2% glucose and 100 µg/ml ampicillin. The next day the library size was determined through colony counting and the bacteria from the 245 mm plates scrapped and inoculated into 8 mls of LB with 8 mls of 40% sterile glycerol. The library was stored in aliquots at −80°C.

### 2.3 Phage Isolation

An aliquot of the phage library was removed from −80°C and inoculated into 60 ml of 2xYT medium with 100 μg/ml ampicillin and 2% glucose. The inoculated medium was grown at 37°C to a 600 nm optical density of 0.4–0.6. Subsequently, 10 ml of the 2xYT culture was transferred to a 50 ml conical tube and 1 μl of CM13 helper phage (Antibody Design Laboratories, San Diego, CA) added with a 1-hour incubation at 37°C in a shaker incubator. Infected cells were pelleted by centrifugation at 2,800 rotational centrifugal force (RCF) for 10 min. Pelleted cells were re-suspended in 50 ml of 2×YT media with 100 μg/ml ampicillin and 50 μg/ml kanamycin and incubated overnight at 30°C in a shaker incubator. The following day, the culture was centrifuged for 15 min at 3,200 RCF to pellet TG1 cells. The media supernatant was decanted into two 50 ml conical tubes, 6 ml of 20% (wt/v) PEG6000/2.5 M NaCl solution were added, and conical tubes were placed in ice for 30-minute. Precipitated phage was pelleted by centrifugation at 10,000 RCF for 20 min. Pelleted phage were re-suspended in 1 ml of PBS and centrifuged for 1.5 min at 16,000 RCF in a microcentrifuge to pellet any residual bacteria. Phage concentration was determined prior to panning *via* a titration method. Briefly, phage was serially diluted by factors of 10 in 2×YT media, and 10 µl of each dilution added to 90 µl of TG1 cells in mid-log phase growth with a subsequent 15-minute incubation at 37°C. Infected TG1 cells from each dilution were spread on LB agar plates with 100 μg/ml ampicillin and 2% glucose and plates incubated overnight at 37°C. Phage concentration was determined by counting colonies on the plate with the highest dilution of phage that grew between 20 and 200 bacterial colonies.

### 2.4 Phage Biopanning

Trastuzumab was chemically conjugated to Dynabeads following manufacturer recommendations (Thermo Fisher Scientific, Waltham, MA). Prior to panning, trastuzumab modified beads were blocked with 2% milk in phosphate buffered saline pH 7.4 (PBS) for 1 h. Two panning strategies were used to isolate low- and high-affinity binders, relative to 1HE. For the first round of panning, the phage library was diluted 100-fold (8.2 × 10^11^ phages/ml) into a 0.2% milk PBS solution and incubated with trastuzumab modified beads for 1 h. Following incubation, beads were washed five times for 5 minutes with PBS. Following washing, a 5 μM solution of 1HE in PBS was added and incubated for 2 h. For the low-affinity panning method, the supernatant, following the 2-hour incubation with 1HE, was removed, and phages amplified in TG1 cells. The panning was repeated identically, with the amplified phage from the previous round for two additional rounds. For the high-affinity panning strategy, following the 2-hour dissociation and supernatant removal, trastuzumab modified beads were incubated with a 100 mM glycine pH 2.0 buffer for 10 minutes. Subsequently, the eluate was removed and neutralized with the addition of 75 μl of 1 M TRIS-HCL pH 9. The output titer was amplified, and two additional pannings performed with a 24-hour and a 72-hour dissociation, for the second and third round, respectively, in a PBS buffer with 5 μM 1HE. The final output titer was infected into TG1 cells, incubated at 37°C for 2 h, with 50 μg/ml ampicillin added 1 h into incubation. Subsequently, phage infected TG1 cells were pelleted by centrifugation, lysed, and phage DNA purified using a plasmid purification kit. Purified phage DNA from the high-affinity panning was transformed into the *E. coli* strain BL21DE3 (New England Biolabs, Ipswich, MA), serially diluted, spread onto LB agar plates with 100 μg/ml ampicillin, and grown overnight at 37°C. Purified phage DNA from the low-affinity panning was digested with XhoI and NdeI restriction enzymes and separated from the Pcomb3XSS plasmid through agarose gel electrophoresis and purified using a gel extraction and purification kit. Low-affinity mutant DNA was ligated into the expression plasmid pET22b(+) (Millipore-Sigma, Burlington, MA) and transformed into the *E. coli* strain SHuffle (New England Biolabs, Ipswich, MA). Transformed cells were spread onto an LB agar plate with 100 μg/ml ampicillin and grown overnight at 30°C.

### 2.5 Phage Screening

A master plate was established by inoculating single SHuffle or BL21DE3 bacterial colonies into the wells of a 96 well plate with 200 μl of LB medium with 100 ug/ml ampicillin, and 20% glycerol and grown overnight at 30°C. The following day, 20 μl of overnight growth media from each well was inoculated into individual wells of a deep well plate with 1 ml of LB, and the starter plate stored at −80°C. The expression plate was grown in a shaker incubator at 300 rpm at 37°C for BL21DE3 or 30°C for SHuffle cells to an optical density at 600 nm of 0.4–0.8. Expression was induced with the addition of 1 mM isopropyl β-d1-thiogalactopyranoside (IPTG) and incubated overnight at 16°C and 300 rotations per minute (RPM). The next day bacterial cells were pelleted by centrifugation at 3,900 RCF for 15 min. Following centrifugation, the culture media was removed, and 100 µl of BugBuster^®^ (Millipore-Sigma, Burlington, MA) with 10 mg/ml lysozyme, and a 1:1,000 dilution of Benzonase^®^ (Millipore-Sigma, Burlington, MA) was added to each well, and bacterial pellets resuspended by pipetting. Cells were incubated with lysis buffer for 15 min at room temperature on a shaker platform at 300 rpm. NUNC Maxisorb plates (Thermo Scientific, Waltham, MA) were incubated with 250 μl of 4 μg/ml trastuzumab in a 20 mM disodium phosphate buffer (pH unadjusted) overnight. The following day, enzyme-linked immunosorbent assay (ELISA) plates were blocked with the addition of 250 μl of a 1% bovine serum albumin (BSA) solution for 1 h. The bacterial lysate from the expression plate was diluted 10-fold in a 0.1% BSA solution, and 25 μl added to individual wells of an ELISA plate containing 225 μl of PBS. The plate was incubated with diluted lysate for 1 h and then washed four times, with 250 μl of phosphate-buffered 0.05% Tween-20 (wash buffer). For the high-affinity clones, ELISA screening was run in duplicate wells. Following binding, a well for each colony was incubated in PBS, and another well for each colony incubated with 1 μM 1HE to prevent rebinding for 40 h. After 40 h, the wells were washed four times with wash buffer and 250 μl of a 1:2,000 dilution of an anti-hemagglutinin alkaline phosphatase (AP) conjugated secondary antibody (Millipore Sigma, Burlington, MA) in 0.1% BSA PBS added to each well and incubated for 1 h on a shaker incubator at 300 RPM. Following incubation, wells were washed three times with wash buffer and two times with distilled water (dH_2_O). 250 μl of 4 mg/ml para-nitrophenyl phosphate (PnPP) in a 10 mM diethanolamine pH 9.8 buffer was added to each well, and the change in absorbance at 405 nm monitored using a SpectraMax 340PC plate reader (Molecular Devices, San Jose, CA) for 10 min at 30-second intervals. An estimate for the trastuzumab binding half-life for each clone was calculated using the half-life equation using the signal from the PBS well as time = 0 and the signal from the well incubated with 1HE as time = 40 h. For the low-affinity panning clones, a similar approach to estimate binding half-life was used. Individual clones were run in triplicate, with a 3-hour and a 6-hour dissociation time point. In addition, 500 μM of trastuzumab was added to block rebinding for the 3- and 6-hour timepoints and a 1:5,000 dilution of an anti-hexahistidine tag AP secondary antibody (Abcam, Cambridge, United Kingdom) used for detection.

### 2.6 Sequencing

Following the screening protocol, 11 Colonies from the low-affinity panning and 13 colonies from the high-affinity panning were selected for DNA sequencing. Mutants were grown in 10 ml of LB media with 100 μg/ml ampicillin overnight at 37°C for BL21DE3 cells or 30°C for SHuffle cells. The next day, cells were pelleted, lysed, and DNA purified using a plasmid purification kit. DNA concentration was determined using a nanodrop and diluted to 100 ng/ml. Low-affinity clones in the Pet22b vector were sequenced using T7 promoter primers, and high-affinity clones were sequenced using pComb3FOR and pComb3REV primers. Sanger sequencing was completed at the Roswell Park sequencing core facility (Buffalo, NY).

### 2.7 Dissociation Rate Constant Screening

1HE mutants were selected from the DNA sequencing results for characterization of the trastuzumab dissociation rate constant. Individual mutants were expressed and purified using a nickel chromatography resin. Nunc Immobilizer Amino (Thermo Fisher Scientific, Waltham, MA) plates were incubated with 100 μl of 5 μg/ml of trastuzumab in a 100 mM disodium phosphate pH 8 buffer overnight. The following day, unreacted sites were blocked with 10 mM ethanolamine in a 100 mM sodium bicarbonate buffer pH 9.5 for 1 h. Purified mutants were incubated for 1 h and subsequently washed three times with wash buffer. Following washing, 0.1% BSA PBS solution was added to the initial timepoint wells. Wells containing the low-affinity mutants were incubated in a buffer with 500 nM trastuzumab, and the high-affinity mutants were incubated in a buffer with 1 μM of purified 1HE. Trastuzumab or 1HE was added to prevent rebinding of the 1HE mutants to trastuzumab, following dissociation. Trastuzumab was included in the low-affinity mutant screening to capture dissociated mutants to prevent rebinding to immobilized trastuzumab. 1HE was used for the high affinity mutant screening to block binding sites on immobilized trastuzumab (following dissociation of 1HE variants). Of note, the high-affinity mutants contained a c-terminal hemagglutinin (HA) tag whereas free 1HE did not; therefore, high affinity mutants bound to trastuzumab could be detected using an anti-HA antibody with dissociated mutants being outcompeted for rebinding to trastuzumab by 1HE. The concentrations of free 1HE and trastuzumab that were spiked into the wells were at a large molar excess (∼100×) relative to immobilized trastuzumab to ensure efficient blockade of dissociated mutant rebinding by either 1) free 1HE outcompeting dissociated high-affinity 1HE mutants rebinding to immobilized trastuzumab or 2) free trastuzumab outcompeting immobilized trastuzumab for rebinding to the low affinity 1HE mutants. At individual time points, the buffer was removed by pipetting, the wells were washed three times with wash buffer, and 0.1% BSA PBS added. At the terminal time point, all wells were washed three times with wash buffer, and secondary antibodies added at the dilutions listed above for 1 h at room temperature. Following secondary incubation, plates were washed three times with wash buffer and three times with dH_2_O. 250 μl of 4 mg/ml PnPP in a 10 mM diethanolamine pH 9.8 buffer was added to each well, and absorbance values read for 10 min at 30-second intervals. The change in absorbance per minute for each well, which is a measure of 1HE mutants bound to immobilized trastuzumab, was divided by the average change in absorbance per minute for the initial time point wells, and the resulting dissociation curves fit to a monoexponential decay function in GraphPad Prism 7 (GraphPad, San Diego, CA). Each time point was run in triplicate for each mutant.

### 2.8 Cell Cytotoxicity Assay

NCI-N87 cells were seeded at a density of 5,000 cells per well of a 96-well U-bottom plate (Corning Inc., Corning, NY). Following overnight incubation, the culture media was removed using a needle vacuum aspiration method and 200 µl of fresh media with a range of T-DM1 concentrations was added to individual wells. Each T-DM1 concentration was run in triplicate on each plate, with plates run in triplicate. Spent media was removed and replaced with fresh T-DM1 dilutions daily for a total treatment duration of 6-days. On the sixth day, media with 1 mg/ml 3-(4,5-dimethylthiazol-2-yl)-2,5-diphenyltetrazolium bromide (MTT) (Sigma, St. Louis, MO) was added to individual wells and incubated for 2 h. Subsequently, 100 ul of a 10% SDS 0.01 M HCl solution was added and incubated overnight to solubilize the formazan crystals. Following solubilization, plates were read at 550 nm to measure formazan dye with normalization at a wavelength of 690 nm using a SpectraMax 340PC plate reader (Molecular Devices, San Jose, CA). The viable cell fraction was determined by dividing the normalized absorbance for T-DM1 treated wells to untreated control wells on the same plate.

### 2.9 Sphere Pharmacokinetic Model Development

A pharmacokinetic model, previously used by our laboratory to predict the effect of anti-vascular endothelial growth factor (VEGF) therapy on topotecan tumor uptake (Shah et al., 2009), was adapted for predicting the impact competitive inhibition would have on trastuzumab and T-DM1 tumor disposition. Systemic concentrations of trastuzumab/T-DM1 and inhibitor were simulated with a 2-compartment model with tumor distribution modeled using a concentric sphere with five well-mixed sub-compartments of equal width (Sphere model). Previously observed plasma pharmacokinetics for trastuzumab and 1HE, in non-tumor bearing mice (Bordeau et al., 2021), were fit to a 2-compartment model using Adapt 5 (BMSR, Los Angeles, CA) (D’Argenio et al., 2009), assuming a mouse bodyweight of 25 g. Model fits for trastuzumab and 1HE are provided in Supplementary Figures 1 and 2 and the fit parameter values and coefficient of variation percentages are provided in Supplementary Table 1. The tumor model represents mAb uptake and distribution from a single tumor blood vessel with an inter-vessel radius (IVR) of 75 μm (Baish et al., 1996; Thurber and Dane Wittrup, 2012). The center compartment of the tumor, layer A, is the point of extravasation for antibody through the vasculature into the tumor space. Upon entry, trastuzumab can bind HER2, diffuse to tumor layer B, or redistribute back into the plasma. Trastuzumab is modeled to enter tumor layers B-E *via* diffusion through connected sub-compartments. Several simplifying assumptions were made for this proof-of-concept investigation, including homogenous tumor antigen expression, no antigen shedding, and diffusion as the only transport mechanism for mAb through the interstitial space. Diffusion was considered to be the only transport mechanism within the tumor interstitial space as high tumor interstitial pressure, resulting from poor lymphatic drainage and high vasculature permeability, eliminates the pressure gradient that drives fluid to flow from the tumor vasculature to the lymphatic system (Jain and Baxter, 1988; Thurber et al., 2008) Tumor layer volume and surface area values were calculated based on the equations for a sphere, assuming a density of 1 g/ml, and can be found in Table 1. For calculating the individual layer volumes, the radius was equal to the width of the layer (15 µm) plus the width of any previous layers. For example the volume of layer C is equal to 4/3 × π × (45 µm)^3^ minus the volume that is accounted for by layers A and B. Trastuzumab permeability, diffusion coefficients, HER2 tumor concentration, and internalization kinetics were obtained from the literature and are provided with references in Table 2. The diffusion coefficient for the inhibitor was calculated using the Stokes-Einstein equation, using the molecular radius of a sdAb (Bhunia et al., 2015), and the vascular permeability coefficient for the inhibitor was based on the permeability of a 25 kilodalton (kDa) antibody fragment (Yuan et al., 1995). The volume transfer rate constant between plasma and Layer A was calculated using the permeability surface area product, with tumor vasculature surface area per unit volume calculated using reported values for tumor blood volumes and the equations for a sphere. Diffusion clearance (CLd) between connected tumor layers was calculated using the equation CLd = D × SA/W where D is the diffusion coefficient, SA the surface area of a tumor layer, and W the width of a tumor layer. The interstitial volume of tumor layers A–E was calculated as the product of the void fraction times the total volume of each layer. To accurately capture target mediated disposition of mAb, plasma exchange with tumor was scaled from a single vessel, represented by the sphere model, to a whole tumor, using the ratio of the whole tumor volume/sphere tumor volume (TVs), as shown in differential equations CP1, CP2, CP3. The value of TVs is time-dependent and increases along with tumor growth. Bivalent trastuzumab binding to 1HE and HER2 was added to accurately capture the impact of 1HE on T-DM1 tumor distribution. Deconjugation clearance (CLdec) of T-DM1 to free trastuzumab was included to capture the decline in the drug antibody ratio (DAR) over time (Bender et al., 2014; Singh et al., 2016). A simplifying assumption was made that deconjugated trastuzumab does not bind free 1HE. To ensure the model was mass-balanced, simulations that included tumor growth included a loss term that was equal to the growth rate for each tumor equation, except for the equation for HER2. All simulations were conducted in Berkley-Madonna Version 9. All model parameters that were calculated can be found in Table 1, and model parameters that were obtained from in-house or previously reported experimental observations can be found in Table 2. Model equations for the systemic pharmacokinetics of T-DM1, 1HE, and trastuzumab and the differential equations for tumor layer A are provided beneath the subsequent methods section. Differential equations for tumor layers B-E are identical in structure to A, apart from there being no antibody exchange with the vasculature and diffusion transport only occurring between connected sub-compartments.

**TABLE 1:** Calculated PK/PD sphere model parameters.

Parameter	Abbreviation	Value	Units	Source
Width of tumor layer	W	1.5E-3	cm	IVR/5
Tumor layer A volume	Va	1.4E-11	L	4/3πW^3^/10^3^
Tumor layer B volume	Vb	9.9E-11	L	4/3π(2W)^3^/10^3^-Va
Tumor layer C volume	Vc	2.7E-10	L	4/3π(3W)^3^/10^3^-Va-Vb
Tumor layer D volume	Vd	5.2E-10	L	4/3π(4W)^3^/10^3^-Va-Vb-Vc
Tumor layer E volume	Ve	8.6E-10	L	4/3π(5W)^3^/10^3^-Va-Vb-Vc-Vd
Interstitial volume A	VaVF	3.4E-12	L	Va×VF
Interstitial volume B	VbVF	2.4E-11	L	Vb×VF
Interstitial volume C	VcVF	6.5E-11	L	Vc×VF
Interstitial volume D	VdVF	1.3E-10	L	Vd×VF
Interstitial volume E	VeVF	2.1E-10	L	Ve×VF
Inhibitor diffusion coefficient	D2	5.1E-2	cm^2^/h	Stokes-Einstein
Vascular volume	VV	2.1E-10	mL	TVF×4/3π(5W)^3^
Vascular radius	VR	3.7E-10	cm	(3/(4π)VV)^1/3^
Vascular surface area	VSA	1.7E-4	cm^2^	4πVR^2^
Vascular PS coefficient TmAb	PSpa	1.7E-10	L/h	P1×VSA/10^3^
Vascular PS coefficient 1HE	PSpa2	2.9E-10	L/h	P2×VSA/10^3^
Surface area layer A	SAa	2.8E-5	cm^2^	4πW^2^
Surface area layer B	SAb	1.1E-4	cm^2^	4π(2W)^2^
Surface area layer C	SAc	2.5E-4	cm^2^	4π(3W)^2^
Surface area layer D	SAd	4.5E-4	cm^2^	4π(4W)^2^
Distribution clearance TmAb A-B	CLdAB	8.9E-9	L/h	D1×SAa/(10^3^×W)
Distribution clearance TmAb B-C	CLdBC	3.5E-8	L/h	D1×SAb/(10^3^×W)
Distribution clearance TmAb C-D	CLdCD	8.0E-8	L/h	D1×SAc/(10^3^×W)
Distribution clearance TmAb D-E	CLdDE	1.4E-7	L/h	D1×SAd/(10^3^×W)
Distribution clearance 1HE A-B	CLdAB2	9.6E-7	L/h	D2×SAa/(10^3^×W)

PS, permeability surface area coefficient; TmAb, trastuzumab.

Volumes A–E represent layer volumes for the disposition model. Volumes A2–E2 represent the starting tumor layer volumes for the tumor growth model for a tumor with a starting volume of 250 mm^3^. VF, D2, P1, P2, IVD values and definitions are provided in Table 2.

**TABLE 2:** Experimentally determined PK/PD sphere model parameters.

Parameter		Value	Units	Source
T-DM1 clearance	CL1	1.0E-5	L/h	—
T-DM1 distribution clearance	CLd1	8.4E-5	L/h	—
T-DM1 central volume	V1	2.1E-3	L	—
T-DM1 peripheral volume	V2	2.2E-3	L	—
1HE clearance	CL2	1.9E-2	L/h	—
1HE distribution clearance	CLd2	5.2E-3	L/h	—
1HE central volume	V3	2.3E-3	L	—
1HE peripheral volume	V4	6.6E-3	L	—
T-DM1 deconjugation	CLdec	1.5E-5	L/h	Bender et al. (2014); Singh et al. (2016)
DM1 Conjugation Ratio	DAR	3.5	—	
kon 1HE:T-DM1	konI	0.89	nM^−1^ h^−1^	Bordeau et al. (2021)
koff 1HE:T-DM1	koffI	0.04	h^−1^	-
MAb diffusion	D1	4.7E-4 (1.7E-4)	cm^2^/h	Berk et al. (1997); Netti et al. (2000); Pluen et al. (2001)
MAb Vasculature permeability	P1	1.0E-3 (4.3E-4)	cm/h	Yuan et al. (1995); Yuan et al. (1996); Dellian et al. (2000)
Vasculature permeability 1HE	P2	1.7E-3	cm/h	Yuan et al. (1995)
Tumor Vasculature Fraction	TVF	0.12 (0.04)	—	Vogel, (1965); Fallowfield, (1989); Baxter et al. (1994); Henderson et al. (2000); Chen et al. (2017)
Intervessel Radius	IVR	75	µm	Cilliers et al. (2016)
Hematocrit	Hct	0.45	—	Green, (1966)
Void Volume Fraction	VF	0.24	—	Schmidt and Wittrup, (2009)
kon Tmab:HER2	konA	2.6	nM^−1^ h^−1^	Bostrom et al. (2011)
koff TmAb:HER2	koffA	1.3	h^−1^	Bostrom et al. (2011)
kon DM1:tubulin	kond	0.018	nM^−1^ h^−1^	Shah et al. (2012)
koff DM1:tubulin	koffd	0.55	1/h	Shah et al. (2012)
Cell Volume	—	5	pL	Erickson et al. (2012); Goldmacher et al. (2015)
DM1 Loss Rate	kloss	0.14	h^−1^	Khera et al. (2018)
Tubulin Concentration	Tub	14,750	nM	Goldmacher et al. (2015)
HER2 internalization rate	kint	4.9E-2 (3.3E-2)	h^−1^	Worthylake et al. (1999); Austin et al. (2004); Maass et al. (2016); Nessler et al. (2020)
HER2 Expression	—	1.5E6 (2.8E5)	Rec/cell	Hendriks et al. (2013); Onsum et al. (2013); Li et al. (2016)
HER2 tumor concentration	Ag0	2075	nM	—
Tumor growth rate	kgex	3E-3 (1E-3)	h^−1^	—
Maximum kill rate constant	Kkill	0.014	h^−1^	Menezes et al. (2020)
DM1 conc. for 50% of Kkill	Km	800	nM	Menezes et al. (2020)
Minimum conc. for killing	—	120	nM	Menezes et al. (2020)
RIT Clearance	—	1.3E-3	L/h	Bauss et al. (2016)
RIT Central Volume	—	9.8E-4	L	Bauss et al. (2016)
RIT Diffusion Rate	—	9.0E-5	cm^2^/h	Chen et al. (2008)
RIT vascular permeability	—	1.8E-3	cm/h	Chen et al. (2008)

Abbreviations: kon, Association rate constant; koff, Dissociation rate constant.

DM1 = T-DM1 catabolites, e.g., lysine-mcc-DM1, conc., concentration; Rec, receptors; IVR, tumor inter-vessel radius.

### 2.10 T-DM1 Pharmacodynamic Model

A mechanism-based pharmacodynamic model was developed to consider tumor cell killing following T-DM1 internalization and degradation. DM1 metabolites transport out of the lysosome and out of the cell with a rate constant (kloss) reported by Khera et al. (2018). In the cell cytosol, DM1 metabolites can bind to tubulin or be effluxed from the cell. Tubulin DM1 binding rate constants were obtained from the report by Shah et al. which was used to characterize MMAE tubulin-binding (Shah et al., 2012); however, the ratio of the koff/kon values (30 nM) is close to the experimentally determined DM1-tubulin equilibrium dissociation constant (K_D_: 11–31 nM) (Goldmacher et al., 2015; Menchon et al., 2018). The intracellular tubulin concentration was obtained from a report by Goldmacher et al. and is an average of four cancer cell lines (Goldmacher et al., 2015). A simplifying assumption was made that DM1 metabolites, which are charged and show poor membrane permeability, do not reflux back into cells. This assumption is consistent with a modeling analysis by Khera et al. that predicted rapid tumor clearance of DM1 metabolites following cellular efflux (Khera et al., 2018). Tubulin bound DM1 drives cell-killing which is modeled using a Hill function, with a maximum killing rate constant (Kkill), a half-maximum concentration (Km) and minimum tubulin bound DM1 concentration to achieve cell-killing of 120 nM as reported by Menzenes et al. (2020). The minimum concentration required for cell-killing was coded using a conditional if then statement with Kkill set to a value of 0 when intracellular DM1 concentrations are less than 120 nM. *In-vitro* cell or tumor growth was included in the model equations with growth causing dilution of bound ADC, or intracellular DM1 concentration. The growth dilution function was set to a value of 0 when the number of cells or tumor volume was less than its initial conditions to prevent artificial concentration of ADC/DM1. An *in vitro* model was developed and predictions for intracellular DM1 catabolites accumulation and ADC catabolism were compared to data reported by Erickson et al. (2012). Data were digitized using WebPlotDigitizer (Ankit Rohatgi, Pacifica, CA, United States, Version 4.5 https://automeris.io/WebPlotDigitizer). Model predictions for NCI-N87 growth and killing *in vitro* were made and compared to the results obtained from the *in vitro* NCI-N87 cell cytotoxicity experiments. Subsequently, the *in vitro* PD model was incorporated into the sphere PK model to predict NCI-N87 xenograft growth/killing following administration of T-DM1. The PK/PD sphere model has two tumor spaces, the first represents T-DM1 disposition around a single tumor vessel, and the second represents tumor growth and killing. The concentration of intracellular DM1 in individual layers of the disposition model drives the killing function in individual layers of the growth model. The initial volumes for each layer in the growth model is the product of the scaling factor (TVs) multiplied by the calculated volume for each layer that is provided in Table 1. Monte Carlo simulations (*n* = 1,000) with variability on the tumor growth rate (mean: 0.003 h^−1^, SD: 0.001 h^−1^, range 0.006–0.00125 h^−1^) and on the initial tumor volume (mean: 250–310 mm^3^, SD: 44 mm^3^, range = 200–400 mm^3^) were completed to predict NCI-N87 tumor volumes at the indicated dosing conditions. Model equations for the pharmacodynamic component of layer A are provided below (CTA12-CTA13, A1).

### 2.11 Systemic Pharmacokinetics

**CP1: Central Compartment Concentration of free T-DM1**

$$\begin{array}{l} \frac{dCP1}{dt}=\frac{Cld}{V1}\times \left(CT1-CP1\right)-\frac{Cl1}{V1}\times CP1-\frac{CLdec}{V1}\times CP1+\\ TVs\frac{PSpa}{V1}\times \left(1-Hct\right)\times \left(CTA1-CP1\right)\\ +koffI\times CP2-konI\times CP1\times CP4\times \frac{V3}{V1} \end{array}$$

**CT1: Peripheral Compartment Concentration of Free T-DM1**

$$\frac{dCT1}{dt}=\frac{Cld}{V2}\times \left(CP1-CT1\right)+koffI\times CT2-konI\times CT1\times CT4\times \frac{V4}{V2}$$

**CP2: Central Compartment Concentration of TDM1-1HE**

$$\begin{array}{l} \;\frac{dCP2}{dt}=\frac{Cld}{V1}\times \left(CT2-CP2\right)-\frac{Cl1}{V1}\times CP2-\frac{CLdec}{V1}\times CP2\\ +TVs\times \frac{PSpa}{V1}\times \left(1-Hct\right)\times \left(CTA2-CP2\right)\\ -koffI\times CP2+koffI\times CP3\\ +konI\times CP1\times CP4\times \frac{V3}{V1}\\ -konI\times CP2\times CP4\times \frac{V3}{V1} \end{array}$$

**CT2: Peripheral Compartment Concentration of TDM1-1HE**

$$\begin{array}{l} \frac{dCT2}{dt}=\frac{Cld}{V2}\times \left(CP2-CT2\right)-koffI\times CT2+koffI\times CT3\\ +konI\times CT1\times CT4\times \frac{V4}{V2}\\ -konI\times CT2\times CT4\times \frac{V4}{V2} \end{array}$$

**CP3: Central Compartment Concentration of TDM1-(1HE)2**

$$\begin{array}{l} \;\frac{dCP3}{dt}=\frac{Cld}{V1}\times \left(CT3-CP3\right)-\frac{Cl1}{V1}\times CP3-\frac{CLdec}{V1}\times CP3\\ +TVs\frac{PSpa}{V1}\times \left(1-Hct\right)\times \left(CTA3-CP3\right)\\ -koffI\times CP3+konI\times CP2\times CP4\times \frac{V3}{V1} \end{array}$$

**CT3: Peripheral Compartment Concentration of TDM1-(1HE)2**

$$\frac{dCT3}{dt}=\frac{Cld}{V2}\times \left(CP3-CT3\right)-koffI\times CT3+konI\times CT2\times CT4\times \frac{V4}{V2}$$

**CP4: Central Compartment Concentration of free 1HE**

$$\begin{array}{l} \frac{dCP4}{dt}=\frac{Cld2}{V3}\times \left(CT4-CP4\right)-\frac{Cl2}{V3}\times CP4\\ +TVs\frac{PSpa2}{V3}\times \left(1-Hct\right)\times \left(CTA4-CP4\right)\\ +koffI\times CP2\times \frac{V1}{V3}+koffI\times CP3\times \frac{V1}{V3}\\ -konI\times CP1\times CP4\times \frac{V1}{V3}\\ -konI\times CP2\times CP4\times \frac{V1}{V3} \end{array}$$

**CT4: Peripheral Compartment Concentration of free 1HE**

$$\begin{array}{l} \frac{dCT4}{dt}=\frac{Cld2}{V4}\times \left(CP4-CT4\right)+koffI\times CT2\times \frac{V2}{V4}\\ +koffI\times CT3\times \frac{V2}{V4}-konI\times CT1\times CT4\times \frac{V2}{V4}\\ -konI\times CT2\times CT4\times \frac{V2}{V4} \end{array}$$

**CP5: Central Compartment Concentration of Trastuzumab**

$$\begin{array}{l} \frac{dCP5}{dt}=\frac{Cld}{V1}\times \left(CT5-CP5\right)-\frac{Cl1}{V1}\times CP5+\frac{CLdec}{V1}\times CP1\\ +\frac{CLdec}{V1}\times CP2+\frac{CLdec}{V1}\times CP3\\ +TVs\frac{PSpa}{V1}\times \left(1-Hct\right)\times \left(CTA-CP5\right) \end{array}$$

**CT5: Peripheral Compartment Concentration of Trastuzumab**

$$\frac{dCT5}{dt}=\frac{Cld}{V2}\times \left(CP5-CT5\right)$$

### 2.12 Tumor Layer A

**CTA1: Concentration of free TDM1 in Layer A**

$$\begin{array}{l} \frac{dCTA1}{dt}=\frac{PSpa}{VaVF}\times \left(1-Hct\right)\times \left(CP1-CTA1\right)\\ +CTA2\times koffI-konI\times CTA1\times CTA4\\ -konA\times CTA1\times CTA6+koffA\times CTA7\\ -\frac{CldAB}{VaVF}\times \left(CTA1-CTB1\right) \end{array}$$

**CTA2: Concentration of free TDM1-1HE in Layer A**

$$\begin{array}{l} \frac{dCTA2}{dt}=\frac{PSpa}{VaVF}\times \left(1-Hct\right)\times \left(CP2-CTA2\right)\\ -CTA2\times koffI+CTA3\times koffI\\ +konI\times CTA1\times CTA4-konI\times CTA2\times CTA4\\ -konA\times CTA2\times CTA6+koffA\times CTA9\\ -\frac{CldAB}{VaVF}\times \left(CTA2-CTB2\right) \end{array}$$

**CTA3: Concentration of free TDM1-(1HE2) in Layer A**

$$\begin{array}{l} \frac{dCTA3}{dt}=\frac{PSpa}{VaVF}\times \left(1-Hct\right)\times \left(CP3-CTA3\right)\\ -CTA3\times koffI+konI\times CTA2\times CTA4\;\\ -\frac{CldAB}{VaVF}\times \left(CTA3-CTB3\right) \end{array}$$

**CTA4: Concentration of free 1HE in Layer A**

$$\begin{array}{l} \frac{dCTA4}{dt}=\frac{PSpa2}{VaVF}\times \left(1-Hct\right)\times \left(CP4-CTA4\right)\\ +CTA2\times koffI+CTA3\times koffI\\ +koffI\times CTA9-konI\times CTA1\times CTA4\\ -konI\times CTA2\times CTA4-konI\times CTA7\times CTA4\\ -\frac{CldAB2}{VaVF}\times \left(CTA4-CTB4\right) \end{array}$$

**CTA5: Concentration of free trastuzumab in Layer A**

$$\begin{array}{l} \frac{dCTA5}{dt}=\frac{PSpa}{VaVF}\times \left(1-Hct\right)\times \left(CP5-CTA5\right)\\ -konA\times CTA5\times CTA6+koffA\times CTA10\\ -\frac{CldAB}{VaVF}\times \left(CTA5-CTB5\right) \end{array}$$

**CTA6: Concentration of free HER2 in Layer A**

$$\begin{array}{l} \frac{dCTA6}{dt}=ksyn-kint\times CTA6-konA\times CTA6\times CTA1-konA\times CTA6\times CTA2\\ -konA\times CTA6\times CTA5-konA\times CTA7\times CTA6-konA\times CTA10\times CTA6+koffA\times CTA7\\ +koffA\times CTA8+koffA\times CTA9+koffA\times CTA10+koffA\times CTA11 \end{array}$$

**CTA7: Concentration of TDM1-HER2 in Layer A**

$$\begin{array}{l} \frac{dCTA7}{dt}=-kint\times CTA7+konA\times CTA6\times CTA1\\ -konA\times CTA6\times CTA7-konI\times CTA7\times CTA4\\ -koffA\times CTA7+koffA\times CTA8\\ +koffI\times CTA9 \end{array}$$

**CTA8: Concentration of TDM1-(HER2)2 in Layer A**

$$\frac{dCTA8}{dt}=-kint\times CTA8+konA\times CTA6\times CTA7-koffA\times CTA8$$

**CTA9: Concentration of TDM1:1HE-HER2 in Layer A**

$$\begin{array}{l} \frac{dCTA9}{dt}=-kint\times CTA9+konA\times CTA6\times CTA2\\ +konI\times CTA7\times CTA4-koffA\times CTA9\\ -koffI\times CTA9 \end{array}$$

**CTA10: Concentration of Trastuzumab-HER2 in Layer A**

$$\begin{array}{l} \frac{dCTA10}{dt}=-kint\times CTA10+konA\times CTA6\times CTA5\\ -konA\times CTA10\times CTA6-koffA\times CTA10\\ +koffA\times CTA11 \end{array}$$

**CTA11: Concentration of Trastuzumab-(HER2)2 in Layer A**

$$\frac{dCTA11}{dt}=-kint\times CTA11+konA\times CTA6\times CTA10-koffA\times CTA11$$

**CTA12: Concentration of intracellular T-DM1 in Layer A**

$$\frac{dCTA12}{dt}=kint\times \left(\frac{VF}{1-VF}\right)\times \left(CTA7+CTA8+CTA9\right)-kloss\times CTA12$$

**CTA13: Concentration of intracellular DM1 in Layer A**

$$\begin{array}{l} \frac{dCTA13}{dt}=kloss\times CTA12\times DAR-kloss\times CTA13\\ -konD\times CTA13\times \left(Tub-CTA14\right)\\ +koffD\times CTA14 \end{array}$$

**CTA14: Concentration of Tubulin bound DM1 in Layer A**

$$\frac{dCTA14}{dt}=konD\times CTA13\times \left(Tub-CTA14\right)-koffD\times CTA14$$

**A1: Volume of Tumor layer A**

$$\frac{dA1}{dt}=kgex\times A1-A1\times \frac{kkill\times \left(CTA14\right)}{Km+\left(CTA14\right)}$$

Initial Conditions CTA6 = Ag0, A1 = Va×TVs, All other differential equations initial conditions = 0.

## 3 Results

### 3.1 1HE Mutants

**FIGURE 1:**
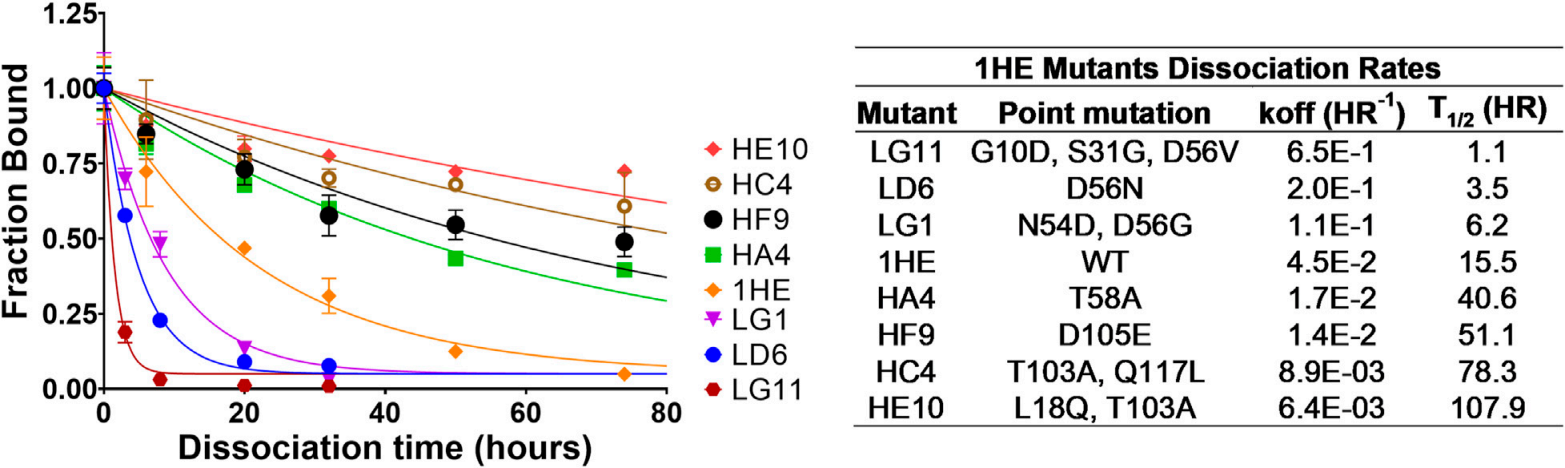
1HE Mutant Trastuzumab Dissociation Rates. (Top left) Plotted is the fractional change in absorbance over time for 1HE mutants that are bound to trastuzumab that is covalently linked to wells of an ELISA plate. Points represent the mean of samples in triplicate with standard deviation error bars. Lines represent the best-fit monoexponential decay for individual mutants. The estimated dissociation rate (koff), the corresponding dissociation half-life and the point mutations relative to wildtype (WT) 1HE for each mutant are provided in the top right inset.

Following panning, 96 colonies from the low-affinity panning and 88 colonies from the high-affinity panning were screened using ELISA to estimate the half-life of trastuzumab binding for each colony. ELISA results were used to select individual colonies over a range of trastuzumab binding half-lives for DNA sequencing. All the sequenced colonies had at least one mutation from the parent sequence of 1HE. Two mutation “hot-spots” were observed for both the low and high-affinity mutants from the parent DNA. Five of the 11 low-affinity mutants had mutations in the center of complementary determining region two (CDR2), with four having mutations at aspartate 56 (D56). Of the thirteen sequenced colonies from the high-affinity panning, four shared an identical mutation of threonine 103 to alanine (T103A) in CDR3. None of the four colonies with the shared T103A mutations had identical sequences, indicating the mutants originated from different parent phages. Three low-affinity and four high-affinity mutants were selected to estimate the dissociation rate constant from trastuzumab using the covalent ELISA method described in the methods section. Dissociation curves and monoexponential decline fittings for individual clones are shown in Figure 1, with clones from the high affinity panning starting with the letter H and clones from the low affinity panning starting with the letter L. Best-fit dissociation rate constants (koff) and estimated half-lives of trastuzumab binding (determined as the quotient of 0.693/koff) are provided in the inset of Figure 1. LG11 with mutations of G10D, S31G, D56V had the fastest dissociation rate with a trastuzumab binding half-life of 1.1 h. HE10 with the mutations L18Q and T103A had the slowest dissociation rate with an estimated trastuzumab binding half-life of 107.9 h. The monoexponential decay function reasonably captured the decline in ELISA signal across timepoints for the mutants. Several early timepoints deviate from the fitting which may be attributed to heterogeneity in either the immobilized trastuzumab (i.e., partially occluded binding sites) or the expressed and purified 1HE mutants (e.g., due to partial degradation, etc.). The present results are consistent with assessments of 1HE and trastuzumab binding and dissociation, as measured by ELISA and surface plasmon resonance, in our prior work (Bordeau et al., 2021). The estimated binding half-life of 1HE for trastuzumab using the covalent ELISA method was 15.5 h, which is close to the value of 12.2 h that was determined using a radiolabeled dissociation method described previously (Bordeau et al., 2021). The covalent ELISA method provides a relative affinity ranking of the 1HE mutants, and further characterization to ensure the accuracy of the estimated dissociation rate and to evaluate potential changes in the trastuzumab association rate constant may be pursued using surface plasmon resonance binding analysis.

### 3.2 Trastuzumab Tumor Uptake

**FIGURE 2:**
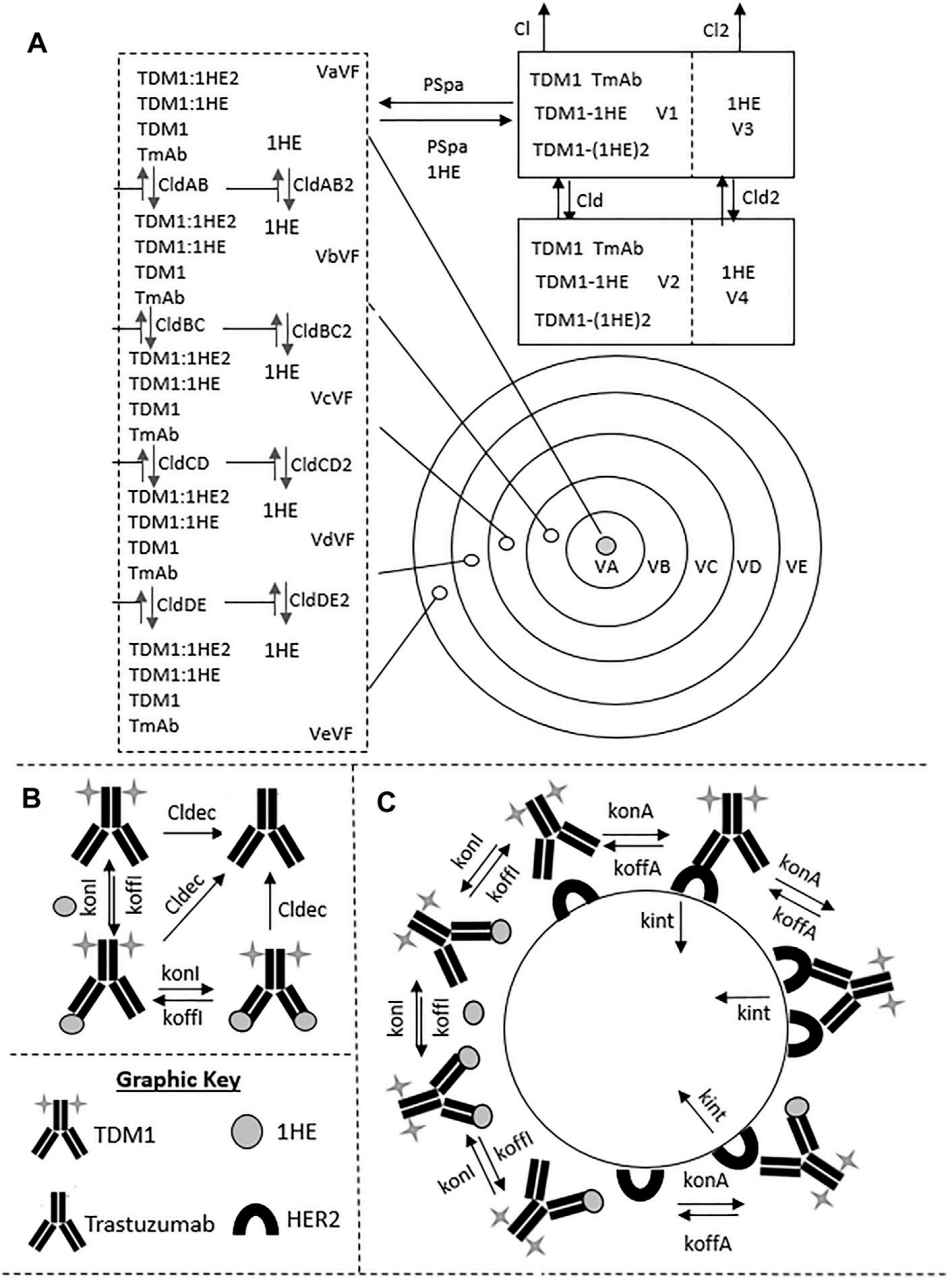
Pharmacokinetic Sphere Model Structure. A graphic representation of the sphere model structure is shown. Panel **(A)** shows the distribution processes of T-DM1, T-DM1:1HE, T-DM1:(1HE)2, 1HE, and Trastuzumab. T-DM1, T-DM1:1HE, T-DM1:(1HE)2, and trastuzumab share distribution parameters from the central and peripheral compartments and share transport rates into and out of the tumor space. Unbound 1HE has unique distribution, clearance, and tumor uptake parameters. Panel **(B)** shows the binding processes between T-DM1 and 1HE that occur within the two-compartment model that represents the systemic disposition of mAb. Shown in section **(B)** is the deconjugation clearance of T-DM1 to free trastuzumab. Panel **(C)** represents the binding processes occurring within each individual tumor layer. Free arms of T-DM1 or T-DM1:1HE can bind free cellular HER2 and can either dissociate or be internalized into the cell layer. Deconjugated trastuzumab can also bind HER2 in each tumor layer but is not explicitly shown due to space limitations. Parameter definitions and values are provided in Tables 1 and 2.

**FIGURE 3:**
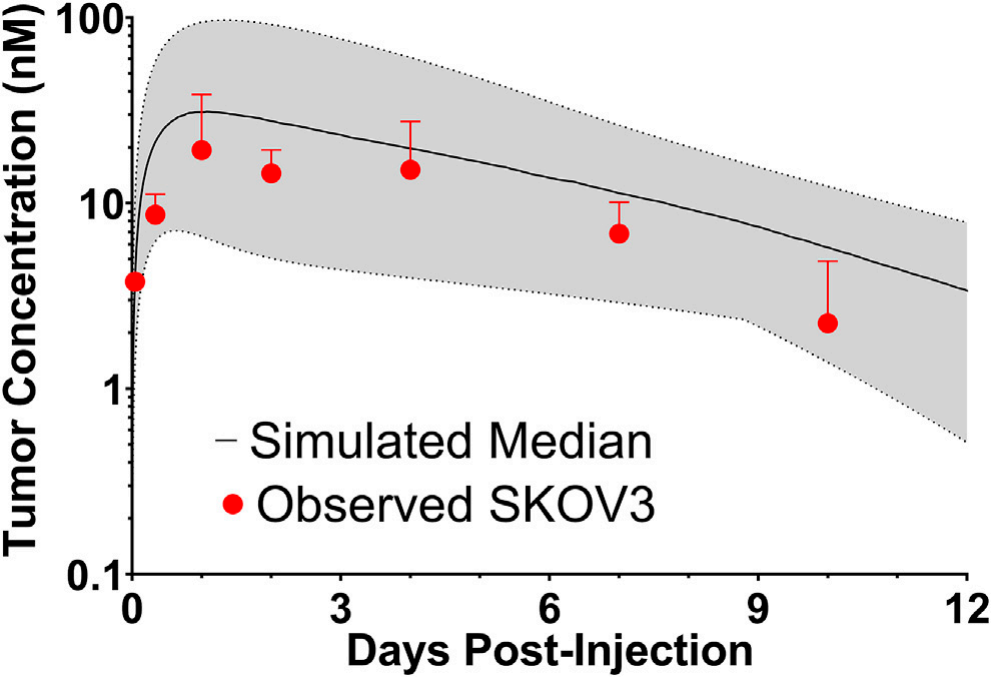
Observed and Sphere Model Predicted Tumor Concentrations. Shown is the median (solid black line) and range (gray shaded region) of 1,000 Monte Carlo simulations for total trastuzumab uptake in HER2+ xenografts following a 1 mg/kg IV bolus. In red is the observed uptake of trastuzumab in SKOV3 xenografts following IV injection of trastuzumab with a tracer dose of ^125^I-trastuzumab. Individual timepoints represent the mean of three xenografts with a standard deviation error bar.

To evaluate if the sphere model structure and parameters accurately characterized total antibody uptake, 1,000 Monte Carlo simulations were completed to predict trastuzumab tumor concentrations following a 1 mg/kg intravenous dose. A schematic representation of the major components of the model is provided in Figure 2. Parameter variability was included on the antibody tumor vasculature permeability rate (mean: 0.001 cm h^−1^, SD: 0.00043 cm h^−1^, range 0.002–0.005 cm h^−1^), tumor blood volume fraction (mean: 0.12, SD: 0.04, range 0.07–0.2), antibody diffusion rate (mean: 0.00047 cm^2^ h^−1^, SD: 0.00017 cm^2^ h^−1^, range 0.0003–0.0007 cm^2^ h^−1^), HER2 antigens per cell (mean: 1.5E6, SD: 2.8E5) and HER2 internalization/degradation rate (mean: 0.049 h^−1^, SD: 0.033 h^−1^, range 0.027–0.12 h^−1^). Our laboratory previously characterized trastuzumab uptake in nude mice bearing HER2+ xenografts of the human ovarian cancer cell line SKOV3 following a 1 mg/kg dose (Abuqayyas and Balthasar, 2012), and this data was used to evaluate model prediction accuracy. The model output for trastuzumab tumor concentration was the sum of both free and HER2 bound trastuzumab in each layer multiplied by the volume of the corresponding layer divided by the total volume of all tumor layers. The median of the Monte Carlo simulations reasonably captured the observed trastuzumab tumor concentration-time profile with a trend for overprediction; however, all observed concentrations fell within the simulation range (Figure 3).

### 3.3 Simulations Predicting the Effect of Co-Administered 1HE on Within-Tumor Distribution of T-DM1

**FIGURE 4:**
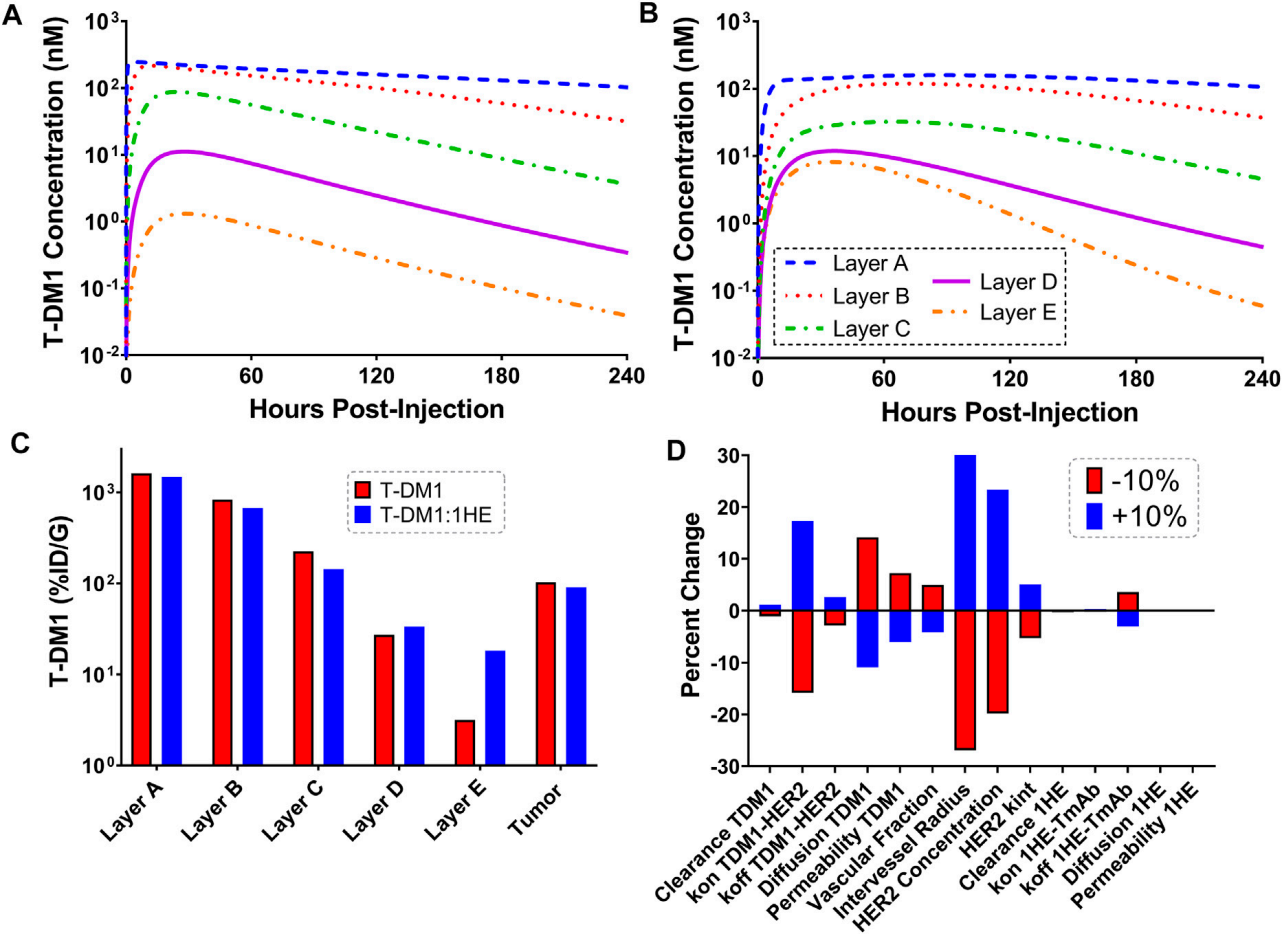
Simulated T-DM1 Distribution with and without 1HE Co-administration. Shown are sphere model simulations for the concentration of T-DM1 bound to HER2 antigen in individual tumor layers over time for T-DM1 administered alone **(A)** or for T-DM1 that is co-administered with 1HE **(B)**. **(C)** Shown is the percent of T-DM1 that is internalized per gram of tumor tissue for each tumor layer with and without 1HE co-administration. **(D)** Sensitivity analysis indicates that the percent of T-DM1 that is internalized in layer E when mAb is administered with 1HE/without 1HE is most sensitive to the intervessel radius, tumor antigen concentration, mAb association rate constant (mAb kon), and mAb diffusion rate constant.

Figures 4A,B show the sphere model predicted concentrations of T-DM1 bound to HER2, in each tumor layer, following T-DM1 administration alone (Figure 4A) or T-DM1 administrated in complex with 1HE (Figure 4B). T-DM1 administered alone leads to maximum concentrations of 246, 216, 88, 11, and 1 nM for tumor layers A-E. T-DM1 administered with 1HE results in maximum concentrations of 160, 119, 32, 12, and 8 nM for tumor layers A-E. Qualitatively, the simulation values are similar to our published experimental observations, where we observed a several-fold increase in trastuzumab fluorescence with 1HE co-administration starting ∼50 µm from the tumor vasculature (Bordeau et al., 2021). Due to the limitations of fluorescence imaging, a direct quantitative comparison of the simulated data to our prior fluorescence imaging data is limited. Figure 4C provides the cumulative total percent of T-DM1 delivered per gram of each tumor layer, which is the percent of T-DM1 eliminated by HER2 internalization in a tumor layer divided by the tumor layer volume in ml. The percent of the injected dose/gram (%ID/G) values for T-DM1/T-DM1:1HE respectively are Layer A: 1,615.4/1,479.2, Layer B: 828.7/673.1, Layer C: 224.0/143.1, Layer D: 27.1/33.4, Layer E: 3.2/18.2, whole tumor: 102.9/90.0. A sensitivity analysis was completed to determine parameters leading to the most significant change in the quotient of T-DM1 delivered to layer E with/without 1HE co-administration (Figure 4D). When adjusted by 10%, parameters representing the tumor tumor inter-vessel radius (IVR), tumor antigen concentration, mAb diffusion rate constant, and mAb association rate constant (kon) led to the largest changes in layer E exposure to T-DM1.

### 3.4 Impact of Inhibitor Dissociation Half-Life on T-DM1 Tumor Distribution

**FIGURE 5:**
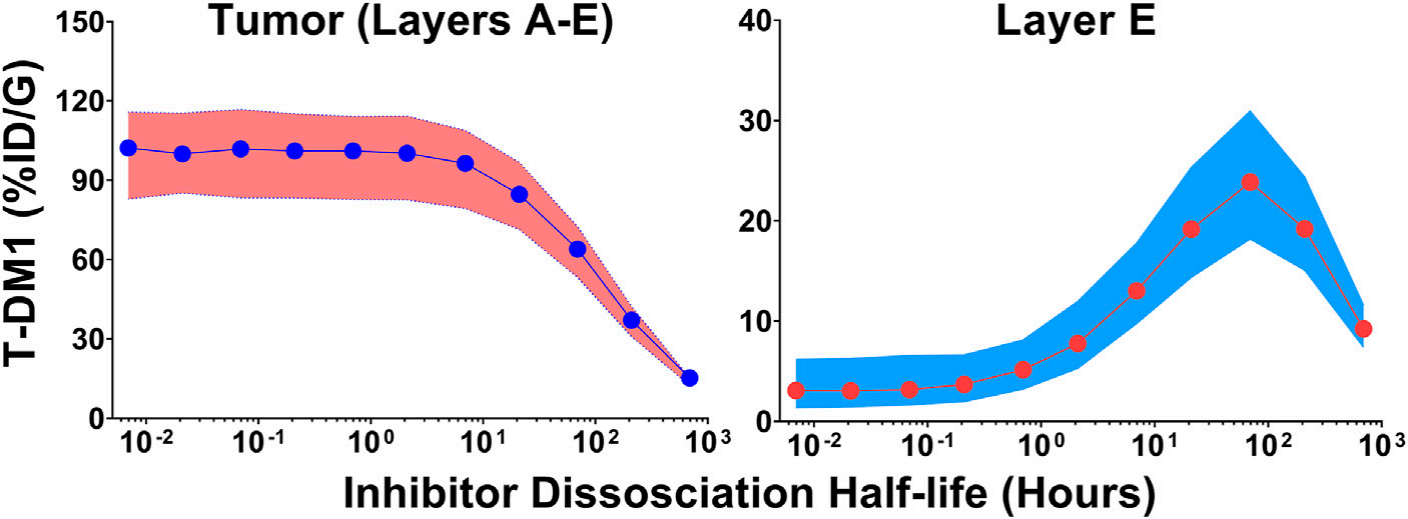
Impact of Inhibitor Binding Half-life on T-DM1 Tumor Distribution. The total percent of T-DM1 that binds to HER2 and is internalized over time, for all tumor layers or for layer E alone, as a function of a co-administered inhibitors half-life is shown. Points represent the median of 1,000 Monte Carlo simulations with the shaded regions representing the first and third quartile of the simulations.

Monte Carlo simulations (*n* = 1,000) were completed across a range of inhibitor dissociation half-lives. Parameter variability was identical to the trastuzumab tumor uptake simulations. Figure 5 shows the cumulative %ID/G of T-DM1 delivered for layers A-E (total tumor uptake) and layer E alone. The total tumor uptake of T-DM1 is negligibly changed by competitive inhibitors with a binding half-life up to ∼10 h, whereas inhibitors with a trastuzumab binding half-life >20 h decrease total tumor uptake by >15%. The median value of total T-DM1 delivered to layer E increases by more than 50% at inhibitor half-lives ≥1 h and reaches a maximum increase of 770% at a half-life of 69.3 h.

### 3.5 *In-Vitro* T-DM1 Efficacy Simulations

**FIGURE 6:**
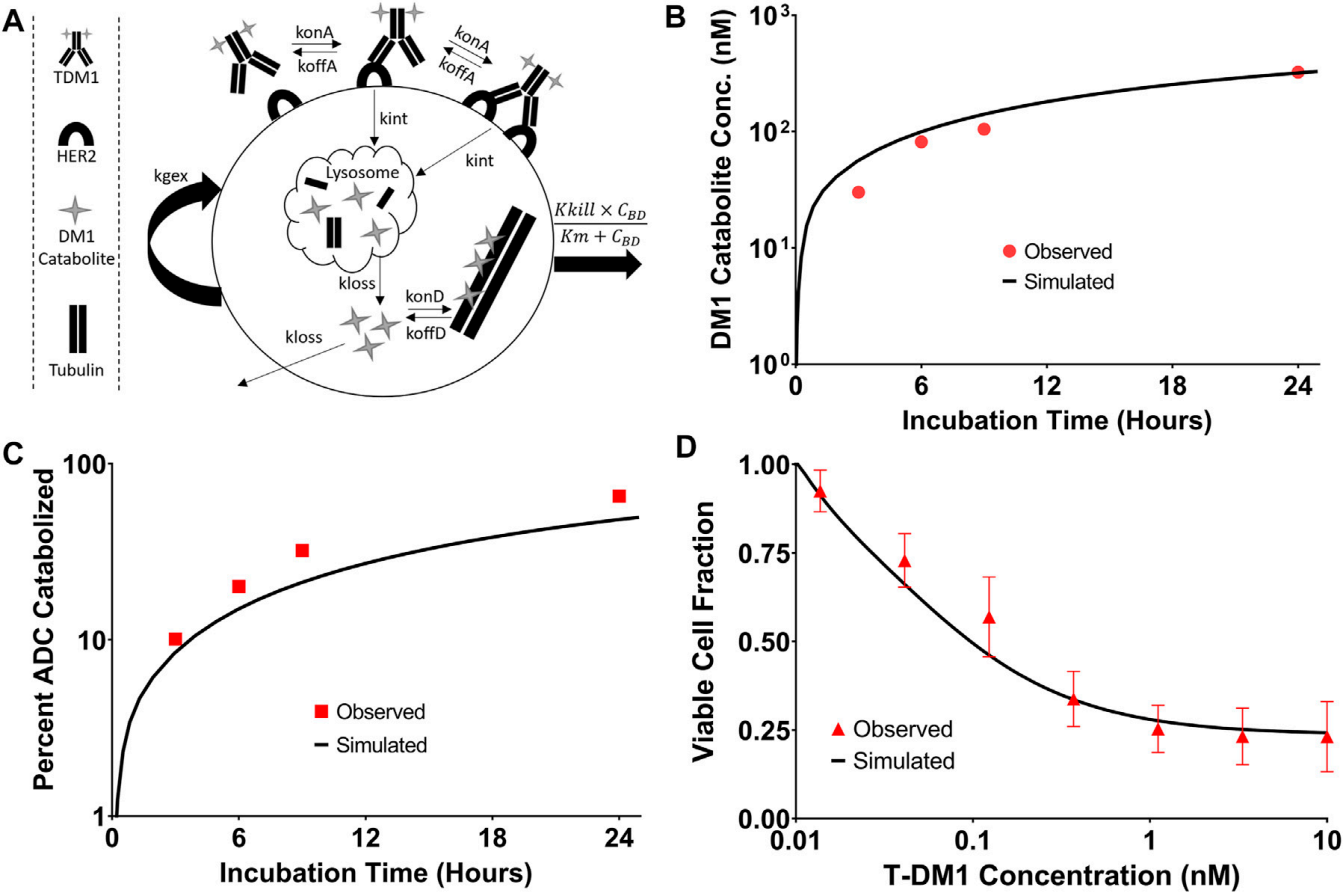
T-DM1 Pharmacodynamic Model: **(A)** A graphic representation of model additions to capture T-DM1 cell killing is provided. Following internalization DM1 catabolites enter the cytosol from the lysosome and either bind to tubulin or are eliminated from the cell. DM1 bound tubulin drives the cell killing hill function. **(B)** represents the *in vitro* model simulations (solid black lines) for accumulation of DM1 catabolites in BT474EEI cells following a 24-hour incubation with T-DM1 in comparison to the data observed by Erickson et al. in red. **(C)** Demonstrates the simulations for the percent of T-DM1 catabolized over time in comparison to the data observed by Erickson et al. **(D)** Model simulations for the fraction of viable NCI-N87 cells treated with T-DM1 in comparison to an untreated control. Red points represent the mean of three assays with standard deviation error bars.

The T-DM1 efficacy model, which makes considerations for DM1-tubulin binding and target cell killing was compared to *in vitro* data to evaluate model accuracy. A schematic of the model structure is provided in Figure 6A. To validate model parameters for T-DM1 metabolism, DM1-tubulin binding, and DM1 cellular efflux, simulated profiles for ADC catabolism and intracellular DM1 concentrations were compared to digitized data reported by Erickson et al. (2012). The simulations captured the observed profiles for DM1 metabolite accumulation (Figure 6B) and percent ADC catabolized (Figure 6C) reasonably well, with a slight overprediction of the DM1 metabolites between 3 and 8 h and an underprediction of the percent of the ADC catabolized. These contradictory results may be due to an unaccounted-for intermediate product (i.e., partially catabolized ADC) that was not included in the model, or not detected by the original authors HPLC assay (Erickson et al., 2012). Target cell killing is included in the model and is driven by a Hill function that is dependent on the concentration of DM1 that is bound to intracellular tubulin. The T-DM1 specific parameter values for Kkill and Km were previously reported by Menezes et al. (2020) and were evaluated in our model structure by comparing predictions that were made using these values and experimental data for NCI-N87 cell growth and killing following T-DM1 treatment. Model simulations for the fraction of viable cells, which is equal to the quotient of the number of cells with T-DM1 treatment divided by the number of cells without treatment, overlaid well with the observed NCI-N87 cytotoxicity data (Figure 6D), supporting the use of these parameter values despite the differences between the model structure that is used here in comparison to the model structure used by Menezes et al. (2020).

### 3.6 *In-Vivo* T-DM1 Efficacy Simulations

**FIGURE 7:**
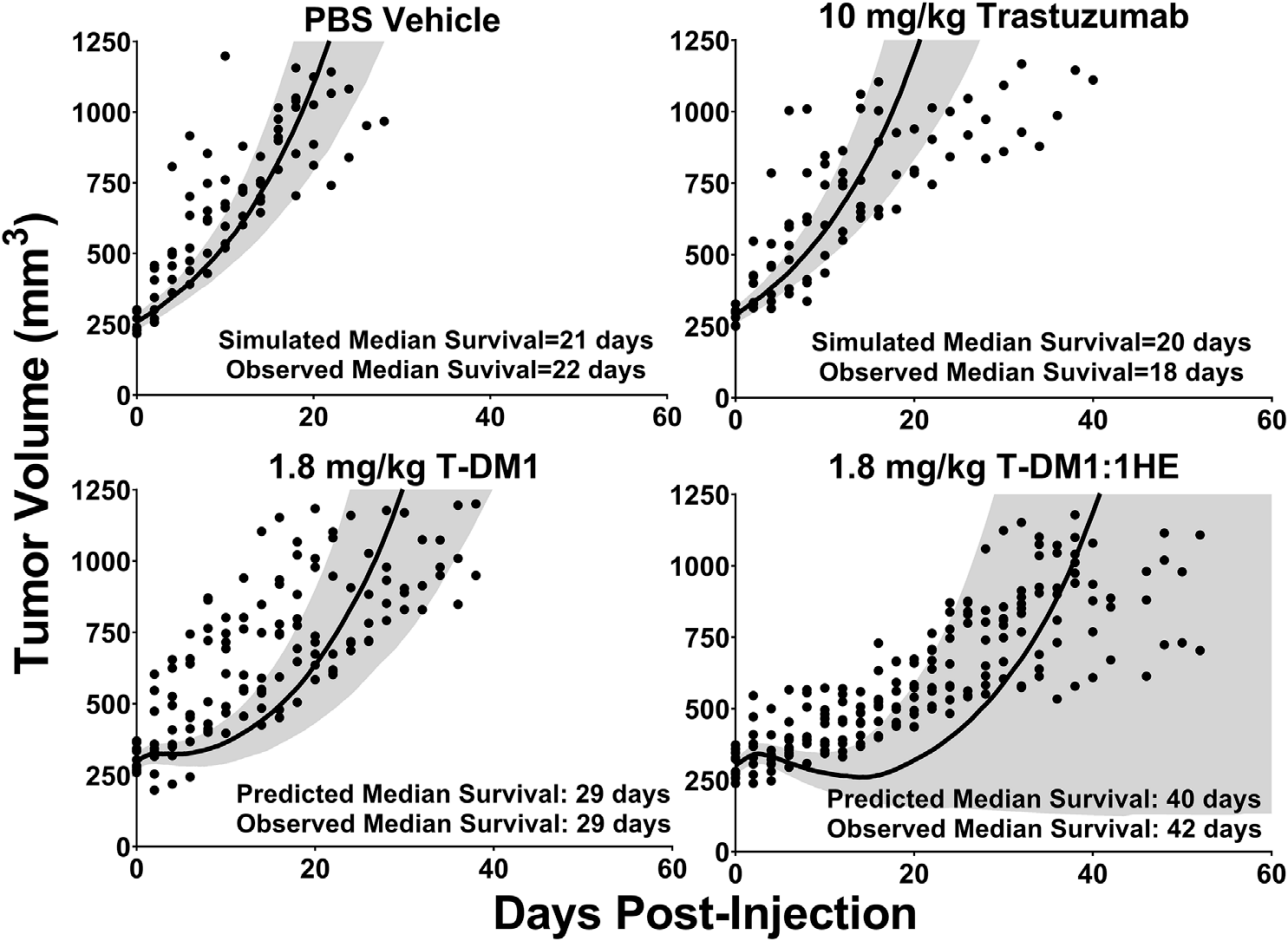
Observed and Predicted Tumor Volumes after a 1.8 mg/kg T-DM1 Dose. PK/PD Model Predictions for the tumor volume of NCI-N87 xenografts over time following a single dose of PBS, 10 mg/kg trastuzumab, 1.8 mg/kg T-DM1, and 1.8 mg/kg T-DM1:1HE. Solid black lines represent the median tumor volume of 1,000 simulations, and the shaded area represents the first and third quartiles. Solid circles represent an individual tumor volume measurement for a single xenograft at one timepoint.

**FIGURE 8:**
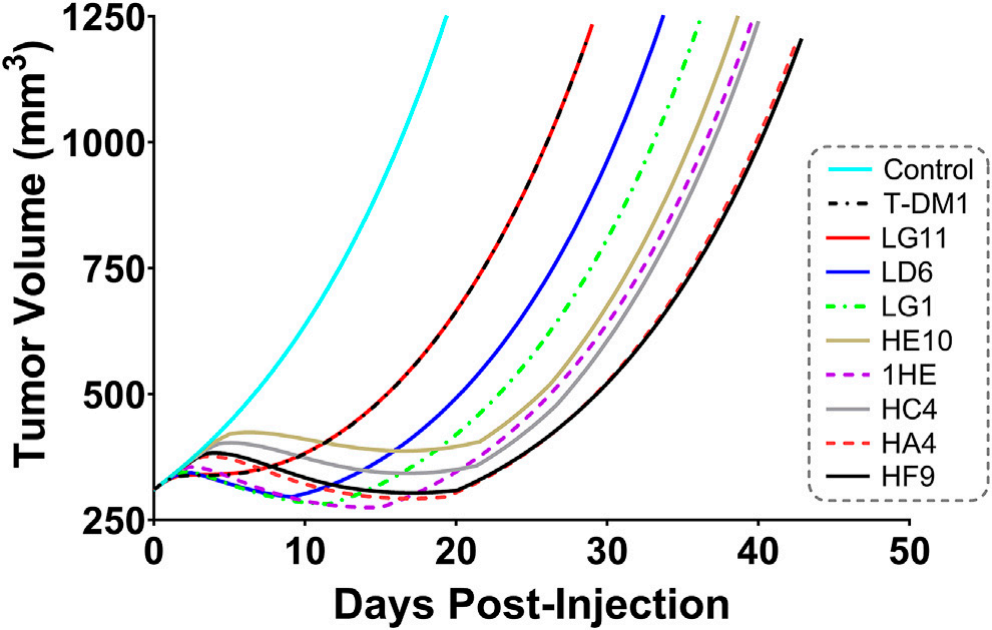
Predicted Impact of 1HE Mutants on T-DM1 Efficacy. PK/PD model predictions for the impact of the 1HE mutants characterized in Figure 1 on T-DM1 efficacy in NCI-N87 xenografts following a single 1.8 mg/kg dose of T-DM1.

Simulations to predict NCI-N87 xenograft tumor volume over time and the median time for tumors to reach a volume of 1,200 mm^3^ (survival time) following an intravenous dose of PBS, 10 mg/kg trastuzumab, 1.8 mg/kg TDM1, and 1.8 mg/kg T-DM1:1HE were completed and compared to our previously observed data (Bordeau et al., 2021). As each tumor layer has unique growth/killing equations the total tumor volume was equal to the sum of the individual simulated tumor layer volumes. 1,000 Monte Carlo simulations with variability on the tumor growth rate (mean: 0.003 h^−1^, SD: 0.001 h^−1^, range 0.006–0.00125 h^−1^) and initial tumor volume (mean: 250–310 mm^3^, SD: 44 mm^3^, range = 200–400 mm^3^) were completed for each dose condition. The mean tumor volume for each dose group was set to the observed mean for the individual groups (PBS control: 255 mm^3^, Trastuzumab: 288 mm^3^, TDM1: 312 mm^3^, TDM1:1HE: 309 mm^3^). Simulation results and observed tumor volumes over time are plotted in Figure 7. Both the PBS and 10 mg/kg trastuzumab simulations accurately captured the observed data, with several of the observed tumor volumes exceeding the 75% quartile at early time points. The median predicted survival time for PBS and trastuzumab was 21 and 20 days, respectively, and the observed median survival time was 22 and 18 days. The predicted 1.8 mg/kg T-DM1 and T-DM1:1HE tumor profiles were well captured, except for several xenografts in the 1.8 mg/kg dose group growing faster than the simulation prediction. The model predicts a greater tumor volume regression for T-DM1 treated tumors approximately 10 days after injection and approximately 15–20 days following T-DM1:1HE administration; however, the median tumor volumes following tumor regression are accurately captured. The simulated median survival time is 29 days for 1.8 mg/kg T-DM1 and 40 days for 1.8 mg/kg T-DM1:1HE, close to the observed times of 29 and 42 days for T-DM1 and T-DM1:1HE, respectively. To predict if any of the 1HE mutants would further enhance T-DM1 efficacy, simulations were completed, without growth variability, using the mutant-specific dissociation rate constants provided in Figure 1. Mutants HA4 and HF9 with trastuzumab binding half-lives of 40.6 and 51.1 h are predicted to modestly increase T-DM1 efficacy, with extensions in the simulated median survival time from 39 days for T-DM1:1HE to 42 days for T-DM1:HA4 or HF9 (Figure 8).

### 3.7 Immunotoxin Simulations

**FIGURE 9:**
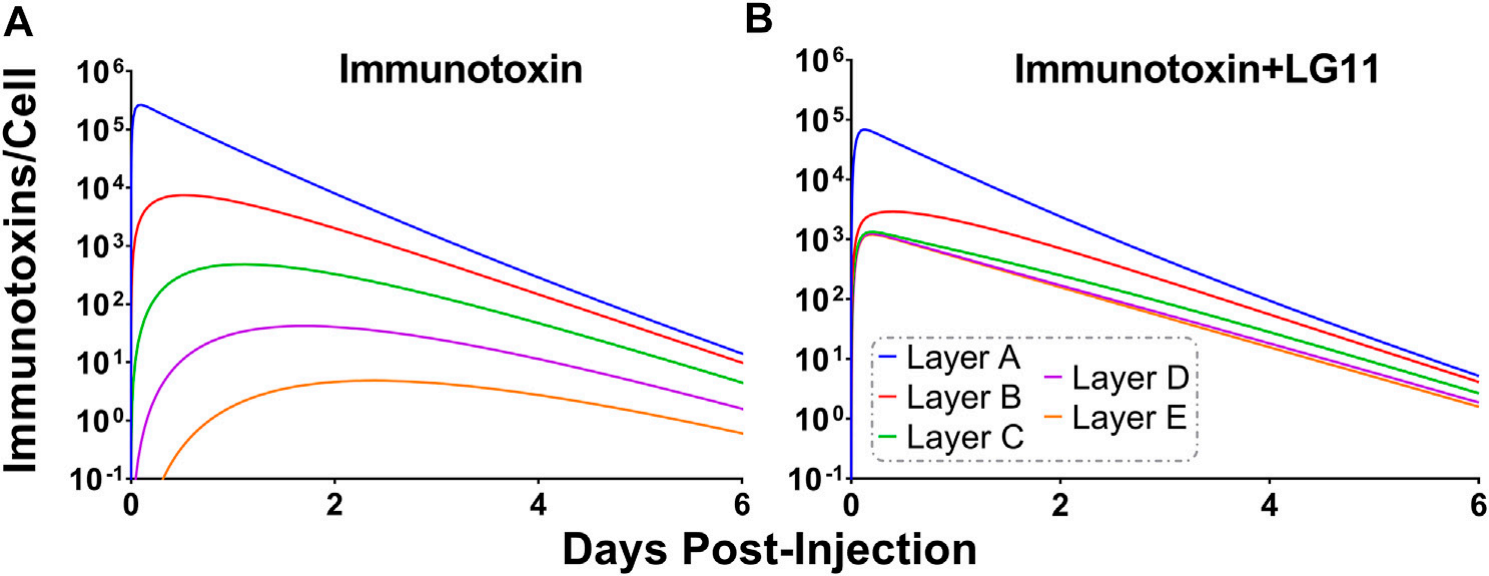
Impact of LG11 on Immunotoxin Tumor Distribution. **(A)** Simulations considering immunotoxin specific pharmacokinetic parameters demonstrate limited tumor penetration following a 0.14 mg/kg dose. Tumor layers C, D, and E are below the 1,000 antigens/cell threshold necessary for tumor cell killing **(B)** Co-administration of immunotoxin with the LG11 mutant (1.1 h trastuzumab binding half-life) results in all tumor layers exceeding 1,000 antigens/cell.

To explore the use of competitive inhibitors for optimizing the within-tumor distribution of immunotoxins, the sphere model was modified with previously reported pharmacokinetic parameters for a recombinant immunotoxin (RIT) (Bauss et al., 2016). Recombinant immunotoxins commonly utilize antibody fragments to target protein toxins, such as pseudomonas exotoxin, to cancer cells (Pastan et al., 2006). Antibody fragments lack the Fc domain of intact antibodies and as a result, have dramatically altered plasma pharmacokinetic profiles. Notably, the clearance value that was used for the RIT is ∼100× greater than the clearance rate that was used for T-DM1. Tumor distribution of a theoretical trastuzumab-based RIT was simulated at a dose of 140 μg/kg, which is the maximum tolerated dose for an anti-mesothelin RIT that is currently being evaluated in clinical trials (Hassan et al., 2020). Prior work has demonstrated that a threshold of ∼1,000 RITs bound per cell is required for cell killing (Kreitman and Pastan, 1998). Figure 9A shows the simulated number of HER2 antigens that are bound by RIT per cell for each layer of the sphere model when RIT is administered alone. Layers A and B exceed the therapeutic threshold, whereas layers C, D and E (representing 93.6% of the total tumor volume) are below the threshold. As a result of the rapid systemic clearance of RIT, co-administration of a high-affinity inhibitor with an inhibition half-life that is much greater than the plasma half-life of RIT would decrease total tumor uptake of RIT, similar to the results observed for T-DM1 in Figure 5. Therefore, we simulated the impact of the fastest dissociating 1HE mutant (LG11) with a binding half-life of 1.1 h (Figure 9B). LG11 co-administration is predicted to increase the number of RITs bound per cell in all tumor layers above the threshold, with layer E predicted to achieve a maximum of ∼1,200 RITs/cell.

## 4 Discussion

In the present work, error-prone PCR and phage display were used to develop and isolate variants of 1HE with trastuzumab dissociation rate constants between 0.0064 and 0.65 h^−1^. The 1HE mutants may be used in strategies to bypass the binding site barrier, enabling improved tumor distribution for a range of trastuzumab-based constructs, and enabling translation of our competitive inhibition strategy beyond pre-clinical animal models. To guide the selection of an optimum inhibitor, simulations were conducted with representation of a solid tumor as a collection of concentric spheres of five sub-compartments. Antibody extravasation within tumors was defined to occur at a peri-vascular site (represented by Layer A in the model), and mAb could then diffuse to distant sites as represented by Layers B-E. Many similar models have been utilized in the past to characterize mAb uptake and penetration into tumors. Jain and Baxter used a spherical model structure to explore the impact of elevated interstitial fluid pressure (IFP) on mAb tumor distribution (Jain and Baxter, 1988). Jain and Baxter predicted that the high IFP in the center of solid tumors restricts mAb extravasation; therefore, most mAb enters the tumor from vessels in the periphery where IFP is lower. As a result, mAb needs to diffuse larger distances than the inter-vessel diameter, which may take many hours or several days (Jain and Baxter, 1988). Fujimori et al. used a sphere model with a well-vascularized outer shell to predict mAb penetration from the outer layer to the tumor core as a function of mAb affinity (Fujimori et al., 1990). This seminal work led to the prediction of the existence of a binding site barrier that limits high-affinity mAb distribution in solid tumors (Fujimori et al., 1990). Significant contributions to the understanding of factors that contribute to the heterogeneous distribution of mAb in solid tumors have been made through a series of publications by the Wittrup group (Graff and Wittrup, 2003; Thurber et al., 2007; Thurber et al., 2008; Thurber and Wittrup, 2008; Schmidt and Wittrup, 2009; Rhoden and Wittrup, 2012; Thurber and Dane Wittrup, 2012). Mathematical predictions combined with experimental studies led to the proposal of two criteria to predict mAb tumor distribution: the clearance modulus and the Thiele modulus (Graff and Wittrup, 2003; Thurber et al., 2008; Thurber and Wittrup, 2008). The clearance modulus is the ratio of the time to saturate tumor antigen to the plasma clearance time (Thurber et al., 2008). The time to saturate tumor antigen, for a high-affinity mAb, is a function of the diffusion rate (or when vessel permeability is rate-limiting, the extravasation rate), inter-vessel radius, and tumor antigen concentration (Thurber et al., 2008). The clearance time is the weighted average half-life of mAb in the plasma (Thurber et al., 2008). The Thiele modulus makes considerations for target-mediated elimination and is the ratio of the time to saturate antigen to the characteristic time of endocytosis (Thurber et al., 2008). mAbs have slow clearance rates, conducive to antigen saturation; however, penetration is significantly hampered by target mediated elimination, with receptor turnover allowing peri-vasculature cells to consume a significant fraction of mAbs that enter the tumor. The sensitivity analysis of the sphere model (Figure 4D) predicts that T-DM1 penetration to layer E is most influenced by inter-vessel distance, tumor antigen expression, antibody diffusion rate, and trastuzumab-HER2 association rate. These results are consistent with expectations based on intuition. The intervessel distance and mAb diffusion rate are determinants of the length of time required for T-DM1 to traverse the intervessel distance. The HER2 concentration and trastuzumab-HER2 association rate constant determine the length of time T-DM1 can diffuse prior to binding HER2. As mAb-antigen binding is a second order process, the interstitial concentration of mAb will also impact the penetration distance. Figure 3 demonstrates that the sphere model accurately characterizes total trastuzumab SKOV3 xenograft tumor concentrations using parameter values obtained across many different xenografts and HER2+ cell-lines. Further improvements in model predictions may be obtained through consideration of cell-line specific HER2 expression/internalization rate and cell-line specific xenograft physiology (e.g., vascularity). Additionally, for some mAbs, the rate of antigen dissociation can significantly impact tumor penetration, as rapidly dissociating mAbs have several chances to diffuse through the tumor interstitial space prior to target mediated elimination. For trastuzumab, sphere model predictions are relatively insensitive to the HER2 dissociation rate constant, indicating that upon binding, a large fraction of HER2 bound trastuzumab is internalized and degraded prior to dissociation.

The initial sphere model simulations were performed to identify an inhibitor dissociation half-life that would improve tumor distribution without significantly impacting the total tumor uptake of T-DM1. The dissociation half-life was considered the prime metric, as the competitive inhibition strategy may be most conveniently applied in the circumstance where the inhibitor can be administered with ADC on the same dosing schedule (i.e., same dosing frequency). Upon administration, inhibitor-bound ADC needs to extravasate and diffuse throughout the tumor tissue prior to inhibitor dissociation. Low-affinity inhibitors, with rapid dissociation rates, do not provide an inhibition window long enough for ADCs to extravasate and diffuse throughout the tumor interstitial space. Conversely, for slowly dissociating inhibitors, the inhibition window can be too long, leading to a significant fraction of ADC being eliminated in complex with an inhibitor, decreasing total tumor uptake. Based on model predictions, inhibitors with a binding half-life between 10 and 70 h (Figure 5) can increase Layer E exposure to T-DM1 by ≥5×, with total tumor uptake decreasing by ≥15% for inhibitor half-lives greater than 20 h. As the distribution benefit gained from a high-affinity inhibitor may supersede any decrease in total tumor uptake, a quantitative framework to evaluate the relationship between T-DM1 distribution and efficacy was required. Toward this aim, the sphere model was adapted to predict the impact of 1HE on T-DM1 efficacy in NCI-N87 xenografts. Significant work has been done to mathematically characterize T-DM1 efficacy by several groups (Singh et al., 2016; Khera et al., 2018; Menezes et al., 2020; Singh et al., 2020), and all of the parameters that were required to modify the sphere model were found in these prior publications. The PK/PD sphere model was able to accurately capture our previously observed efficacy data for T-DM1 and T-DM1:1HE at a single dose of 1.8 mg/kg. The best 1HE mutant for increasing T-DM1 efficacy was predicted to be HF9 (Figure 8), with an inhibition half-life of 51.1 h. This result highlights the need to consider a dynamic component to modeling and simulation as HF9 is predicted to decrease total tumor uptake of T-DM1 by >25%. When T-DM1 is administered alone, perivasculature tumor regions are exposed to T-DM1 in excess of that necessary to achieve a therapeutic effect, therefore the dose of T-DM1 that is “wasted” due to the overkilling effect is predicted to have a greater impact on overall efficacy than an inhibitor with a long half-life of binding, such as HF9, that may decrease total tumor uptake. Notably, when T-DM1 is administered alone, ∼50% of the total T-DM1 delivered to the tumor is accounted for by tumor layers A and B despite these layers representing only 6.4% of the total tumor volume. The model-based predictions that suggest that an 8% increase in median survival may be obtained with HF9 co-administration with T-DM1, relative to 1HE, were not experimentally validated. A follow up study to validate the model simulations would require a group size of ∼300 mice to achieve a statistical power of 80% to detect the 8% predicted increase in median survival time given the ∼30% variability in NCI-N87 xenograft tumor growth.

Although model predictions indicate that the 1HE mutants we identified here would not substantially improve T-DM1 efficacy in xenograft-bearing mice, relative to 1HE, we believe these mutants will be superior to 1HE when applied in other circumstances. For example, in humans, the plasma half-life of antibodies and ADCs are several-fold longer than in mice. In addition, human tumors are less vascularized (Lauk et al., 1989) and have greater extracellular matrix development (Cybulska et al., 2018) in comparison to mouse xenograft models. Therefore, a longer window of inhibition may be necessary to allow antibodies to extravasate and diffuse into human tumors. A circumstance in which an inhibitor with a shorter binding half-life may be beneficial is in the case of a potent trastuzumab conjugate with rapid plasma elimination. For example, a single-chain variable fragment of trastuzumab has been used to target the ribosome-inactivating protein, pseudomonas exotoxin (Sokolova et al., 2017; Lee et al., 2019). Immunotoxin conjugates have short plasma half-lives (∼1 h) and are highly cytotoxic, with approximately 1,000 bound molecules leading to cell death (Kreitman and Pastan, 1998; Bauss et al., 2016). Sphere model simulations using previously reported plasma pharmacokinetics of an immunotoxin in mice (Bauss et al., 2016) indicate a 1HE mutant with a binding half-life of ∼1 h would decrease the dose required to achieve the 1,000 bound antigens/cell threshold in layer E of the sphere model by ∼50-fold. As immunotoxins commonly have dose-limiting toxicities (Alewine et al., 2015), the competitive inhibition strategy is likely to be facilely scaled to improve immunotoxin efficacy. Interestingly, a theoretical modeling analysis by Pak et al. predicted that shed antigen may improve recombinant immunotoxin efficacy, as shed-antigen bound immunotoxin can penetrate deeper into solid tumors (mechanistically analogous to the competitive inhibition approach described here) (Pak et al., 2012). To experimentally validate model predictions, the efficacy of an anti-mesothelin immunotoxin was compared in tumor models with low and high rates of mesothelin shedding. In contrast to model predictions, immunotoxin efficacy was decreased in the high shed antigen model (Awuah et al., 2016). The decreased efficacy may result from enhanced plasma elimination of shed mesothelin-bound immunotoxin. Enhanced plasma elimination of mAb, when bound to a soluble antigen, has been observed previously by our group, with co-administration of recombinant carcinoembryonic antigen (CEA) leading to a ∼2-fold increase in plasma elimination of the anti-CEA mAb T84.66, decreasing total tumor exposure of T84.66 in a CEA positive xenograft mouse model by 55% (Abuqayyas, 2012). Enhanced elimination of anti-CEA mAb was also reported recently by Iwano et al., with the observation that mAb engineered to have preferential binding for membrane CEA, can in part, overcome shed antigen mediated elimination (Iwano et al., 2019). Additionally, the recombinant immunotoxins utilized to validate model simulations were reported to have a half-life of approximately 1 h (Bauss et al., 2016), therefore, shed antigen which is continuously being produced may lead to a significant fraction of immunotoxin being eliminated in the inhibited form, decreasing total tumor cell uptake. Shed HER2 was not included in the sphere model, however, it would be interesting to extend the model to evaluate any relationship between HER2 shedding and the impact of competitive inhibitors on trastuzumab tumor disposition.

The 1HE mutants identified in the current study span a wide range of dissociation rates constants; however, additional constructs with specific binding affinities or enhanced stability may be necessary. Only a fraction of the total clones from the low- and high-affinity master plates were chosen for DNA sequencing, and only a fraction of the clones that were sequenced were characterized using the covalent ELISA dissociation assay. Therefore, many additional clones with unique sequences and unique trastuzumab binding affinities are likely to be found among our unscreened colonies. The sequences that have been identified, and characterized, provide information on the paratopes of 1HE that are responsible for trastuzumab binding. The center of CDR2 appears to be critical for high-affinity binding to trastuzumab, as most of the clones with faster dissociation rates have mutations at ^59^NGDST^63^. Alvarez-Rueda et al. reported the CDR2 residues ^59^NGDST^63^ as being similar to the HER2 motif ^571^NGS^573^, which is part of a region of HER2 that is bound by trastuzumab (Alvarez-Rueda et al., 2009). Therefore, if necessary, 1HE mutants with faster dissociation rate constants may be rationally designed through site-directed mutagenesis at the ^54^NGDST^58^ motif of CDR2. Alvarez-Rueda et al., also reported that the CDR3 sequence ^103^ TGDGHRADY^111^, showed identity to the HER2 epitope ^575^ TCFGPEADQ^583^ at four positions; however, based on the crystal structure of trastuzumab in complex in with HER2, only the A and D residues are likely to interact with trastuzumab (Alvarez-Rueda et al., 2009). Consistent with the A and D residues being critical for trastuzumab binding, none of the sequenced colonies had mutations at these residues. Threonine 103 was observed to be a common mutation site among the mutants with slower dissociation rate constants, relative to wild-type 1HE. Specifically, six mutants had mutations of threonine to alanine, serine, or isoleucine. Identification of these critical mutation “hot-spots” will support the rational design of competitive inhibitors or allow informed selection of 1HE colonies for further characterization, following DNA sequencing.

In the present work, we have developed competitive inhibitors of trastuzumab: HER2 binding with binding dissociation half-lives between 1.1 and 108 h. These constructs will be used to further the development of our competitive inhibition strategy to bypass the binding site barrier. Here we demonstrate that modeling and simulation using mechanistic models can be used to support the rationale selection of a competitive inhibitor for optimization of trastuzumab-cytotoxin conjugate efficacy.

## Data Availability

The raw data supporting the conclusions of this article will be made available by the authors, without undue reservation.
