# Supplementary material for: Development and Evaluation of Competitive Inhibitors of Trastuzumab-HER2 Binding to Bypass the Binding-Site Barrier
Source: Front Pharmacol. 2022 Feb 18;13:837744. doi: 10.3389/fphar.2022.837744 (PMC8895951; doi:10.3389/fphar.2022.837744)
Supplement: Supplementary file 1 [file DataSheet1.docx]

**Supplementary Information**

**Development and Evaluation of Competitive Inhibitors of Trastuzumab-HER2 Binding to Bypass the Binding-Site Barrier**

Brandon M. Bordeau^1^, Lubna Abuqayyas^1^, Toan D. Nguyen^1^, Ping Chen^1^, & Joseph P. Balthasar^1^

^1^Department of Pharmaceutical Sciences, School of Pharmacy and Pharmaceutical Sciences, University at Buffalo, Buffalo, NY 14214

**Correspondence**

Joseph Balthasar

jb@buffalo.edu


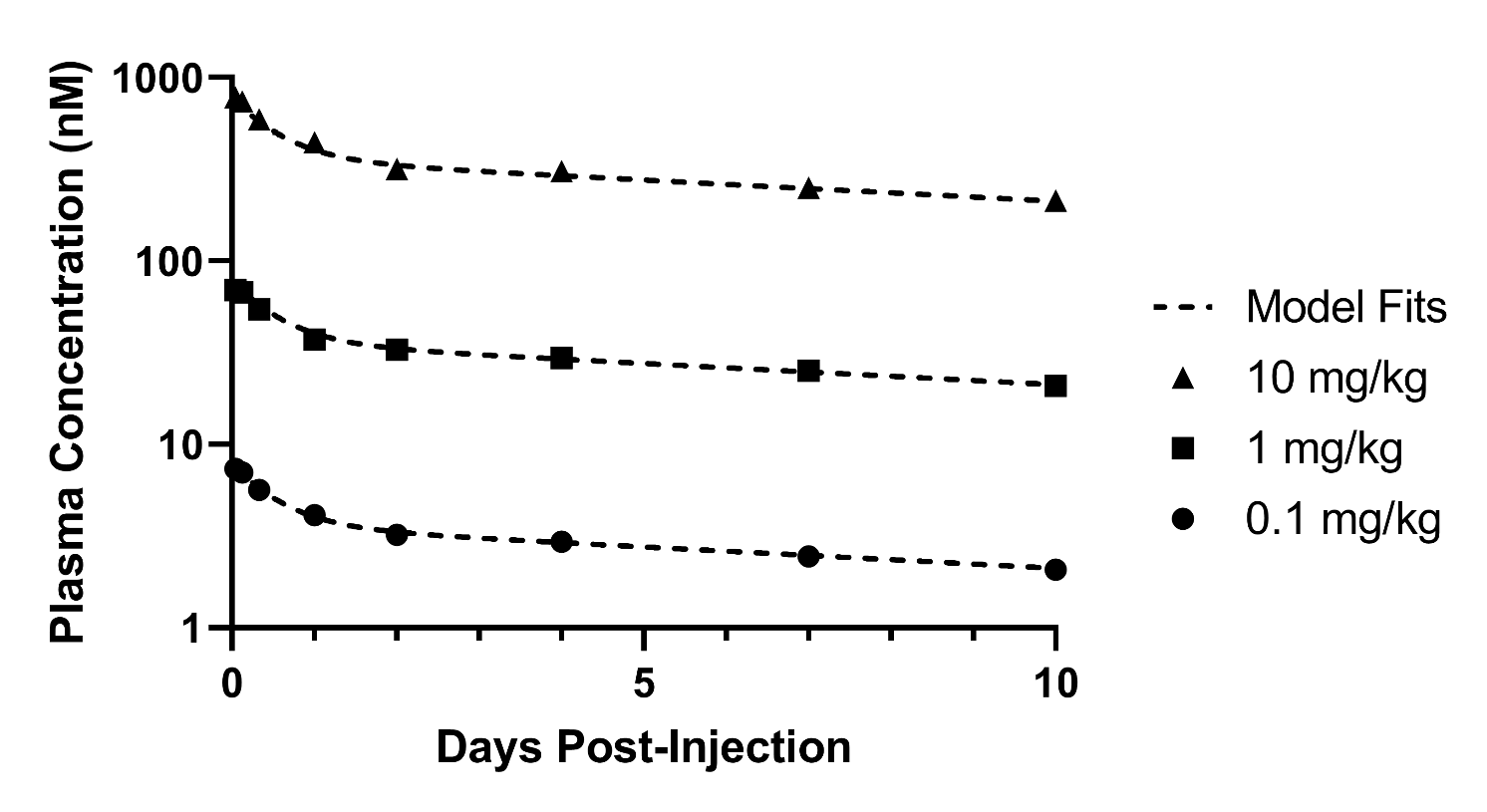


**Supplementary Figure 1:** Shown are the observed plasma concentrations of trastuzumab overtime in Swiss-Webster mice following a single intravenous dose at 10, 1 and 0.1 mg/kg. Observed data was fit to a 2-compartment model in Adapt 5 and fit parameter values used for the 2-compartment structure of the sphere model shown in figure 2. Fit parameter values are provided in supplementary table 1. Additional information (quantitative methods, timepoints, etc) about the trastuzumab pharmacokinetic study can be found in our prior work [1]


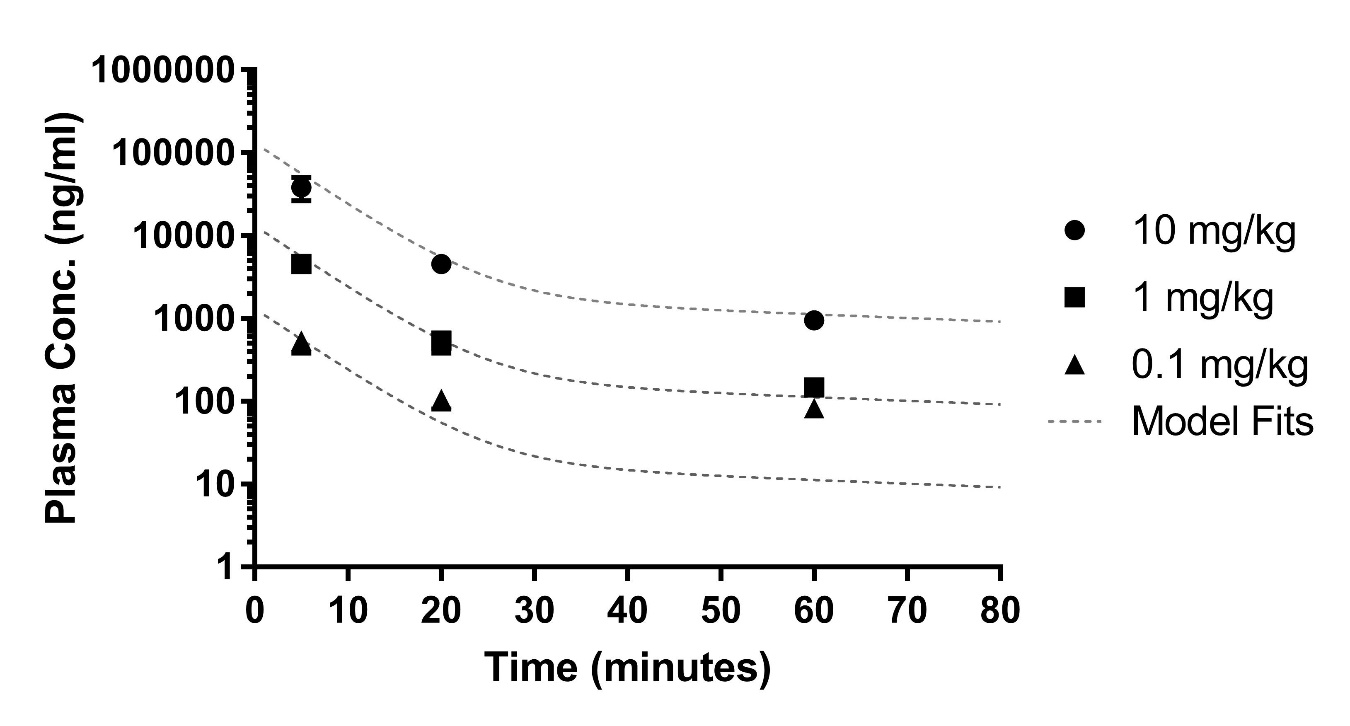


**Supplementary Figure 1:** Shown are the observed mean plasma concentrations of 1HE overtime in Swiss-Webster mice following a single intravenous dose at 10, 1 and 0.1 mg/kg. Observed data was fit to a 2-compartment model in Adapt 5 and fit parameter values used for the 2-compartment component of the sphere model structure that is shown in figure 2. Fit parameter values are provided in supplementary table 1. Additional information (quantitative methods, timepoints, etc) about the 1HE pharmacokinetic study can be found in our prior work [1]

| **Supplementary Table 1: 2-Compartment Model Fittings** | | | |
| --- | --- | --- | --- |
| **PK Parameter** | **Fit Value** | **Units** | **CV (%)** |
| **1HE Cl** | **18.03** | **L/(daykg)** | **3.47** |
| **1HE Cld** | **4.94** | **L/(daykg)** | **0.29** |
| **1HE Vc** | **0.09** | **L/kg** | **4.85** |
| **1HE Vp** | **0.26** | **L/kg** | **0.26** |
| **Trastuzumab Cl** | **9.57E-03** | **L/(daykg)** | **5.82** |
| **Trastuzumab Cld** | **8.08E-2** | **L/(daykg)** | **9.36** |
| **Trastuzumab Vc** | **8.54E-02** | **L/kg** | **2.15** |
| **Trastuzumab Vp** | **8.81E-01** | **L/kg** | **5.58** |

**REFERENCES**

[1] B.M. Bordeau, Y. Yang, and J.P. Balthasar, Transient Competitive Inhibition Bypasses the Binding Site Barrier to Improve Tumor Penetration of Trastuzumab and Enhance T-DM1 Efficacy. Cancer Res 81 (2021) 4145-4154.
